# Gut-derived bacterial flagellin induces beta-cell inflammation and dysfunction

**DOI:** 10.1080/19490976.2022.2111951

**Published:** 2022-08-19

**Authors:** Torsten P.M. Scheithauer, Hilde Herrema, Hongbing Yu, Guido J. Bakker, Maaike Winkelmeijer, Galina Soukhatcheva, Derek Dai, Caixia Ma, Stefan R. Havik, Manon Balvers, Mark Davids, Abraham S. Meijnikman, Ömrüm Aydin, Bert-Jan H. van den Born, Marc G. Besselink, Olivier R. Busch, Maurits de Brauw, Arnold van de Laar, Clara Belzer, Martin Stahl, Willem M. de Vos, Bruce A. Vallance, Max Nieuwdorp, C. Bruce Verchere, Daniël H. van Raalte

**Affiliations:** a Department of (Experimental) Vascular Medicine, Amsterdam UMC, University of Amsterdam, Amsterdam, The Netherlands; b Diabetes Center, Department of Internal Medicine, Amsterdam, The Netherlands; c Department of Pediatrics, Division of Gastroenterology, Hepatology and Nutrition, and BC Children’s Hospital Research Institute, Vancouver, British Columbia, Canada; d Departments of Surgery and Pathology and Laboratory Medicine Pathology and Laboratory Medicine, BC Children’s Hospital Research Institute, Centre for Molecular Medicine & Therapeutics, Vancouver, British Columbia, Canada; e Department of Public and Occupational Health, Amsterdam UMC, University of Amsterdam, Amsterdam, The Netherlands; f Department of Surgery, Amsterdam UMC, University of Amsterdam, Cancer Center Amsterdam, the Netherlands; g Department of Surgery, Spaarne Gasthuis, Hoofddorp, The Netherlands; h Laboratory of Microbiology, Wageningen University and Research, Wageningen, The Netherlands; i Human Microbiome Research Program, Faculty of Medicine, University of Helsinki, Helsinki, Finland

**Keywords:** Gut microbiota, type 2 diabetes, inflammation, beta-cell function, flagellin

## Abstract

Hyperglycemia and type 2 diabetes (T2D) are caused by failure of pancreatic beta cells. The role of the gut microbiota in T2D has been studied, but causal links remain enigmatic. Obese individuals with or without T2D were included from two independent Dutch cohorts. Human data were translated *in vitro* and *in vivo* by using pancreatic islets from C57BL6/J mice and by injecting flagellin into obese mice. Flagellin is part of the bacterial locomotor appendage flagellum, present in gut bacteria including Enterobacteriaceae, which we show to be more abundant in the gut of individuals with T2D. Subsequently, flagellin induces a pro-inflammatory response in pancreatic islets mediated by the Toll-like receptor (TLR)-5 expressed on resident islet macrophages. This inflammatory response is associated with beta-cell dysfunction, characterized by reduced insulin gene expression, impaired proinsulin processing and stress-induced insulin hypersecretion *in vitro* and *in vivo* in mice. We postulate that increased systemically disseminated flagellin in T2D is a contributing factor to beta-cell failure in time and represents a novel therapeutic target.

## Introduction

While obesity is linked to insulin resistance, it is failure of pancreatic beta-cells that drives hyperglycemia and subsequent type 2 diabetes (T2D)^1^. Although in later stages of T2D insulin secretory rates are lowered, prior to the diagnosis and in earlier phases of the disease, insulin secretion is actually increased.^2^ Insulin hypersecretion, particularly in the fasted state, is considered harmful as it is associated with impaired proinsulin processing, insulin secretory stress and depletion of intracellular insulin stores,^3^ further promoting obesity and T2D development.^4^ Drivers of hyperinsulinemia are still elusive but could relate to islet-exposure to excessive nutrients such as carbohydrates and lipids,^5^ as well as a chronic low-grade inflammatory response known to be present in beta cells of people with T2D.^6^ In this regard, an influx of pro-inflammatory macrophages in islets of people with T2D has been noted.^7^ These macrophages produce pro-inflammatory cytokines such as interleukin (IL)-1β and IL-6, which have been associated with insulin hypersecretion^8^ and beta-cell failure.^7^ The triggers that ignite beta-cell inflammation in T2D remain presently unknown.

A known player in the field of glucose metabolism is the intestinal microbiota.^9^ Several cohort and intervention studies have shown an association between gut microbiota composition and T2D incidence.^10^ Individuals with obesity and T2D often have lower microbial diversity while showing increased abundance of potentially pathogenic gram-negative bacteria, including Proteobacteria.^11^ Mechanistic studies have linked metabolites produced by the gut microbiota to impaired glucose metabolism and a pro-inflammatory state.^12^ In addition to microbial metabolites, structural components of gram-negative bacteria, such as lipopolysaccharide (LPS), a cell-wall component, and flagellin, a structural protein of the bacterial locomotor appendage flagellum, may systemically disseminate in people with T2D.^13^ These bacterial molecules activate pro-inflammatory pathways by binding to pattern-recognition receptors (PRRs), including Toll-like receptors (TLRs), expressed on epithelial cells and cells of the innate immune system.^9^

Here, we provide evidence for a novel pathway in which exaggerated systemic dissemination of gut-derived flagellin in T2D induces a pro-inflammatory state in beta cells. This inflammatory response is mediated by flagellin-mediated activation of TLR5 expressed on resident islet macrophages. Functionally, the inflammatory response is associated with impaired insulin gene expression and proinsulin processing while inducing hyperinsulinemia. Collectively, these processes markedly reduce insulin storage, which potentially contribute to beta-cell failure over time.

## Results

### Fecal *Enterobacter cloacae* abundance is associated with hyperglycemia in humans

**Figure 1.**
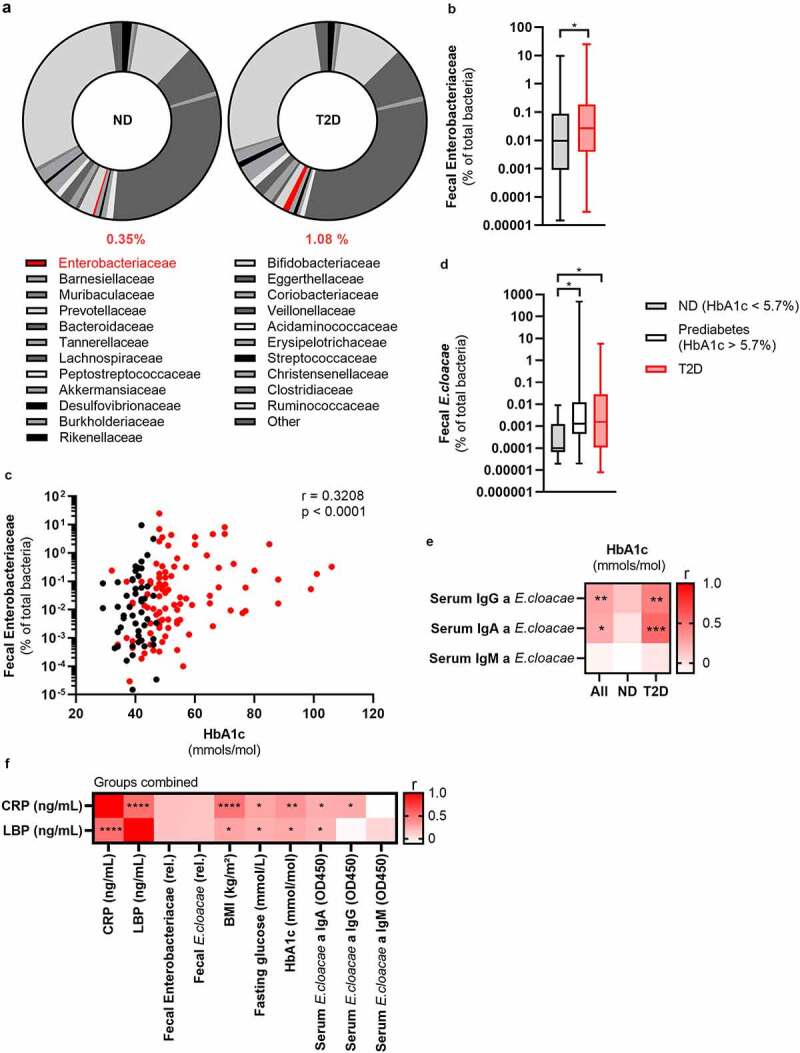
Fecal Enterobacteriaceae correlates with glucose values in the HELIUS cohort.

To investigate the link between beta-cell dysfunction and altered gut microbiota, we analyzed fecal samples for microbiota composition using 16S rRNA sequencing in participants enrolled in the Healthy Life in an Urban Setting (HELIUS) study, a prospective cohort in Amsterdam.^14^ To prevent confounding effects of ethnic differences on gut microbiota composition,^15^ we analyzed the samples of 803 Dutch origin participants (**Table S1**). We observed increased abundance of Gram-negative Enterobacteriaceae in people with T2D as compared to normoglycemic controls (Figure 1a**, Table S2**), confirming a previous report where Enterobacteriaceae were increased in people with T2D.^16^ Detailed compositional analysis can be found in Balvers *et al*.^17^ To reduce the influence of confounding factors, we randomly selected 100 people with T2D and compared them to 50 age-, sex- and BMI-matched normoglycemic controls also recruited within the HELIUS cohort (**Table S3**). We confirmed an enrichment of Enterobacteriaceae in people with T2D using quantitative polymerase chain reaction (qPCR) (Figure 1b). Furthermore, we observed a positive relation with the long-term glucose marker hemoglobin A1c (HbA1c) and Enterobacteriaceae abundance (Figure 1c).

The fecal 16s rRNA of the gut bacteria from the HELIUS cohort was sequenced and quantified via qPCR. Further, the long-term glucose marker HbA1c and antibodies against *Enterobacter cloacae* were measured. (a) Fecal microbiota composition of people with or without T2D was measured via 16S rRNA sequencing (all Dutch origin participants, N = 803, % abundance, median). See table S2 for detailed composition. Here, Enterobacteriaceae is highlighted in red. (b) Fecal Enterobacteriaceae (qPCR, normalized to total fecal bacterial DNA) is increased in people with T2D compared to age-BMI-sex matched healthy controls (N = 150, box plot with min and max). (c) Fecal Enterobacteriaceae (qPCR, normalized to total fecal bacteria) positively correlates with the long-term glucose marker hemoglobin A1c (HbA1c) (N = 150). (d) Fecal *Enterobacter cloacae* (qPCR, normalized to total fecal bacteria) is increased in prediabetes and T2D (N = 150, box plot with min and max). (e) Correlation analysis of serum antibodies against *E. cloacae* and HbA1c (N = 80). (f) Correlation analysis of inflammatory markers CRP and LBP compared to various characteristics (N = 80). Mann Whitney test (b, d) and Spearman correlation (c, e); *p < .05, **p < .01, ***p < .001. Abbreviations: ND, no diabetes; T2D, type 2 diabetes; HbA1c, glycated hemoglobin; Ig, immunoglobulin; CRP, C-reactive peptide; LBP, Lipopolysaccharide binding protein.

*Enterobacter cloacae (E. cloacae)*, a prominent member of the family of Enterobacteriaceae, was previously shown to be associated with impaired glucose tolerance in humans and mice.^18^ In line with these studies, in our cohort, levels of *E. cloacae* directly increased with deterioration of glucose tolerance (Figure 1d). Further, fecal abundance of *E. cloacae* also positively correlated with HbA1c (**Figure S1A**). Thus, as a proof-of-concept, we selected *E. cloacae* for subsequent experiments although we acknowledge that other bacteria of the family Enterobacteriaceae may also associate with glucose (dys)metabolism.

### An immune response against *Enterobacter cloacae* is associated with hyperglycemia in people with type 2 diabetes

An appropriate immune response to opportunistic bacteria is necessary to prevent inflammation. To assess whether there was a systemic immune response to *E. cloacae*, we measured plasma antibody levels. We observed a numerical increase in IgG titers against *E. cloacae* in T2D, but otherwise no significant difference between the matched groups with respect to antibodies was noted (**Figure S1B**). Since obesity *per se* is a known driver of gut dysbiosis and impaired immune response to antigens, it may explain why we found no differences between the two groups.^19,20^ However, there was a positive correlation between HbA1c levels and systemic IgG as well as IgA against *E. cloacae* (Figure 1e), particularly in people with T2D. Further, there was a significant positive correlation between fecal abundance of Enterobacteriaceae and plasma IgG against *E. cloacae* (**Figure S1C**). Lastly, C-reactive peptide (CRP) and LPS binding protein (LBP) positively correlated with glucose values, BMI and antibodies against *E. cloacae*. Further, there was a trend towards a positive correlation between fecal Enterobacteriaceae and *E. cloacae* with LPB and CRP (p < .18). This is suggestive of an immune response against systemically disseminated bacterial components of *E. cloacae*.

### *Enterobacter cloacae* induces beta-cell inflammation and dysfunction *in* *vitro*

**Figure 2.**
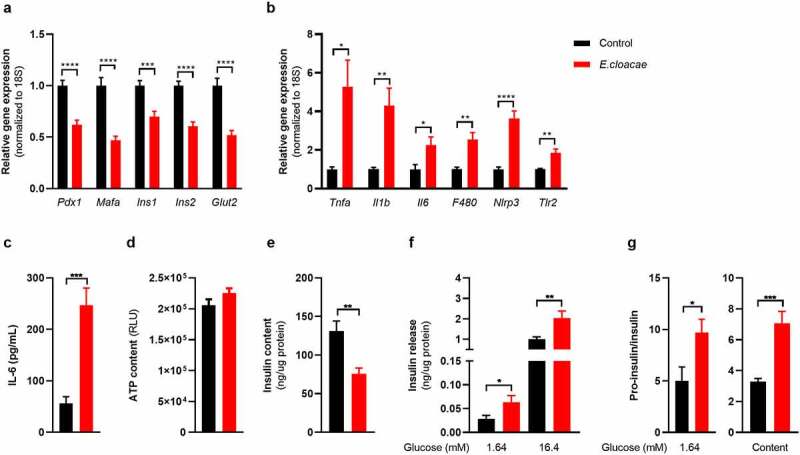
*Enterobacter cloacae* induces beta-cell inflammation and dysfunction.

Given the link between beta-cell driven hyperglycemia and fecal presence of *E. cloacae* as well as systemic antibodies against *E. cloacae*, we questioned if *E. cloacae* would be able to alter pancreatic beta-cell function. We isolated pancreatic islets from C57BL6/J mice fed a conventional chow diet. Islets were co-incubated with 10^6^ colony forming units (CFUs) of heat-inactivated *E. cloacae* or vehicle for 72 hours (Figure 2a-g). We found that beta-cells exposed to heat-inactivated *E. cloacae* had lower expression of genes involved in insulin production, including the key transcription factors pancreatic duodenal homeobox 1 (*Pdx1*) and *Mafa* (Figure 2a). This lowered expression coincided with a higher inflammatory tone (Figure 2 b and c), including upregulation of pro-inflammatory cytokines (*Il1b, Il6* and tumor necrosis factor *α*), the *NLRP3* inflammasome, the macrophage marker *F4/80*, and *Tlr2*. Interestingly, increased *Tlr2* expression was previously reported in pancreatic islets of people with diabetes.^21^ Heat-inactivated *E. cloacae* did not affect cell viability since ATP content was not altered (Figure 2d).^22^ Incubation with heat-inactivated *E. cloacae* also had functional consequences for beta cells. As such, insulin content was markedly reduced after 72 hours of incubation with *E. cloacae* (Figure 2e) but was unchanged for pro-insulin content (**Figure S2A**). In addition, both during low- and high glucose concentrations, beta cells treated with heat-inactivated *E. cloacae* increased insulin secretion (figure 2f). Lastly, *E. cloacae* treatment increased fasting proinsulin secretion as well as fasting proinsulin/insulin ratios, indicating disturbed proinsulin processing (Figure 2g**; Figure S2B**). This highlights that insulin expression and processing is disturbed potentially due to reduced expression of important transcription factors such as *Pdx1* and *Mafa* involved in the insulin production (Figure 2a). We observed similar data in human islets, where heat-inactivated *E. cloacae* lowered *MAFA* expression, increased secreted IL-6, and tended to reduce insulin content (**Figure S2C-G**). Thus, the profile of increased inflammation, reduced insulin gene expression, impaired proinsulin processing and insulin hypersecretion potentially contributes to the detrimental reduction in beta-cell insulin content.

Freshly isolated pancreatic islets from healthy C57BL6J mice were treated with heat-inactivated bacteria for 72 h (10^6^ colony forming units/mL). (a) *Enterobacter cloacae* reduces expression of beta-cell genes, (b) increases expression of inflammatory genes in islets, (c) increases IL-6 secretion by islets, (d) does not change ATP content in islets, (e) reduces insulin content in islets, (f) increases insulin secretion from islets during low- and high glucose conditions, (g) increases the ratio between secreted pro-insulin and insulin, as well as the islet content of pro-insulin relative to insulin. Data shown are mean ± SEM (three representative experiments per panel, n = 9). Unpaired t-test (a, b, c, d, g) and Mann Whitney test (e, f) were used. Significance level: *p < .05, **p < .01, ***p < .001, ****p < .0001. Gene expression was normalized using *18s* as a reference gene. Abbreviations: PDX1, pancreatic and duodenal homeobox 1; INS1 and INS2, insulin 1 and 2; NLRP3, NACHT, LRR and PYD domains-containing protein 3; TNF-α, tumor necrosis factor-alpha; IL-1β, Interleukin 1 beta; IL-6, Interleukin 6; TLR2, Toll-like receptor 2; RLU, Relative light unit.

### Opportunistic bacteria, but not beneficial bacteria, induce beta-cell inflammation and dysfunction

Next, to address whether the *E. cloacae*-mediated effects were specific to this bacterial species, we repeated the experiments with *Escherichia coli (E. coli)*, another Gram-negative bacterium from the family Enterobacteriaceae.^23^ *E. coli* was also increased in T2D participants of the HELIUS study (**Figure S1D**). In line with our *E. cloacae* findings, *E. coli* reduced expression of insulin genes (**Figure S3A**) and induced an inflammatory response with increased expression of *Il6* (**Figure S3B**), and IL-6 protein secretion (**Figure S3C**). *E. coli* also reduced cellular insulin content (**Figure S3D**) and increased insulin secretion (**Figure S3E-F**). In order to rule out an effect of bacterial co-incubation *per se*, we investigated the effects of two bacteria that have been identified as beneficial for the host. These included the Gram-positive *Faecalibacterium prausnitzii*^16^ and Gram-negative *Bacteroides ovatus*.^24^ The abundance of *F. prausnitzii* was decreased in people with T2D (**Figure S1F**). In contrast to the two opportunistic bacteria, *F. prausnitzii* and *B. ovatus* did not affect islet inflammation, insulin content or insulin secretion (**Figure S3A-F**). These data indicate that only a subset of bacteria induces an inflammatory response and beta-cell dysfunction. Based on previous mouse data linking *E. cloacae* to impaired glucose tolerance,^18^ we decided to further scrutinize the effect of this bacterium on beta-cell function as proof-of-concept.

### Toll-like receptor-2 and Toll-like receptor-4 deletion do not protect against *Enterobacter cloacae*-induced beta-cell inflammation and dysfunction

TLR2 and TLR4 are involved in beta-cell replication^21^ and have been proposed as two key PRRs that mediate the inflammatory response induced by endogenous and exogenous molecules, the latter including bacterial components such as LPS.^25^ In addition, TLR2 and TLR4 are expressed by pancreatic islet cells.^26^ Therefore, we isolated islets from TLR2 and TLR4 knockout mice and incubated them with *E. cloacae* (**Figure S4**). Despite the absence of TLR2, *E. cloacae* reduced insulin gene expression (**Figure S4A**), increased expression of pro-inflammatory cytokines (**Figure S4B**), increased secreted IL-6 (**Figure S4C**), and reduced insulin content (**Figure S4D**). Similarly, TLR4-deficient islet cells were not protected from the effects of *E. cloacae*, as the expression of insulin genes was still reduced (**Figure S4F**). Regarding inflammation, *E. cloacae* incubation did not increase *Il6* expression but secretion was still increased despite TLR4 knockout (**Figure S4G-H**). Insulin content was also reduced by *E. cloacae* incubation (**Figure S4I**). Therefore, we concluded that PRRs other than TLR2 and TLR4 likely play roles in *E. cloacae*-induced beta-cell inflammation and dysfunction.

### Toll-like receptor-5 deletion protects against *Enterobacter cloacae*-induced beta-cell dysfunction

**Figure 3.**
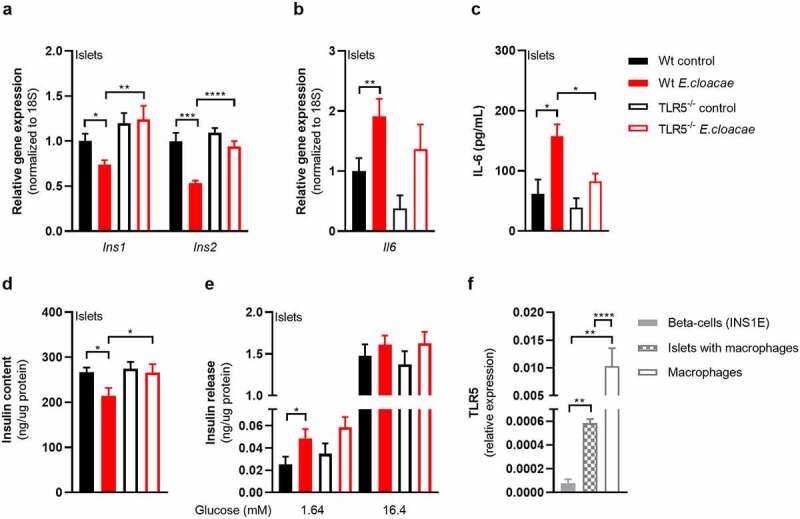
TLR5 mediates beta-cell dysfunction in pancreatic islets.

Several members of Enterobacteriaceae, including *E. cloacae*, express flagellins as both virulence and motility factors.^27^ Bacterial flagellin is recognized by TLR5,^28^ which is expressed by various cell types including epithelial cells and monocytes. We measured the effects of *E. cloacae* in islets from TLR5 knockout mice. TLR5 deletion partially protected islets from beta-cell dysfunction with preserved expression of insulin genes (Figure 3a). TLR5 deficiency did not reduce the effects of *E. cloacae* on the expression of *Il6* (Figure 3b), although it did reduce the secretion of IL-6 as compared to WT islets (Figure 3c). In addition, in TLR5 knockout islets, *E. cloacae* did not reduce insulin content (Figure 3d), while similar insulin secretion rates were observed as from WT islets (Figure 3e).

Freshly isolated pancreatic islets from C57BL6J TLR5^−/−^ mice (10 weeks old) were treated with heat-inactivated*Enterobacter cloacae* (10^6^ CFUs/mL) for 72 h. (a) TLR5 knockout protects from *E. cloacae*-induced insulin expression loss, (b) prevents the effect of *E. cloacae* on pancreatic islet inflammation, (c) partially protects from *E. cloacae* induced IL-6 secretion, (d) protects from *E. cloacae*-induced insulin content loss. (e) *E. cloacae* induces insulin hypersecretion at low glucose condition in wild-type islets. (f) Clonal beta cells express low levels of TLR5 compared to pancreatic islets and macrophages. Data shown are mean ± SEM (three representative experiments per panel, n = 9). Unpaired t-test (a-c, f) and Mann Whitney test (d, e) were used for statistical analysis. Significance level: *p < .05, **p < .01, ***p < .001, ****p < .0001. Abbreviations: INS1 and INS2, insulin 1 and 2; IL-6, Interleukin 6; TLR5, Toll-like receptor 5.

To further assess the role of TLR5 in mediating the effects of *E. cloacae*, we co-incubated WT primary mouse islets with *E. cloacae* with or without the TLR5 inhibitor TH1020. As TH1020 proved toxic to cells after prolonged incubation, we studied the islets after 6h of treatment. In line with TLR5 knockout islets, TH1020 reduced the effects of *E. cloacae* on insulin gene expression and partially reduced the *E. cloacae*-induced expression of *Il6* (**Figure S5A-B**). Due to the toxic effects of TH1020, particularly on *Glut2* expression, we did not measure glucose-stimulated insulin secretion.

### Macrophages mediate *Enterobacter cloacae*-induced beta-cell inflammation and dysfunction via TLR5 activation

**Figure 4.**
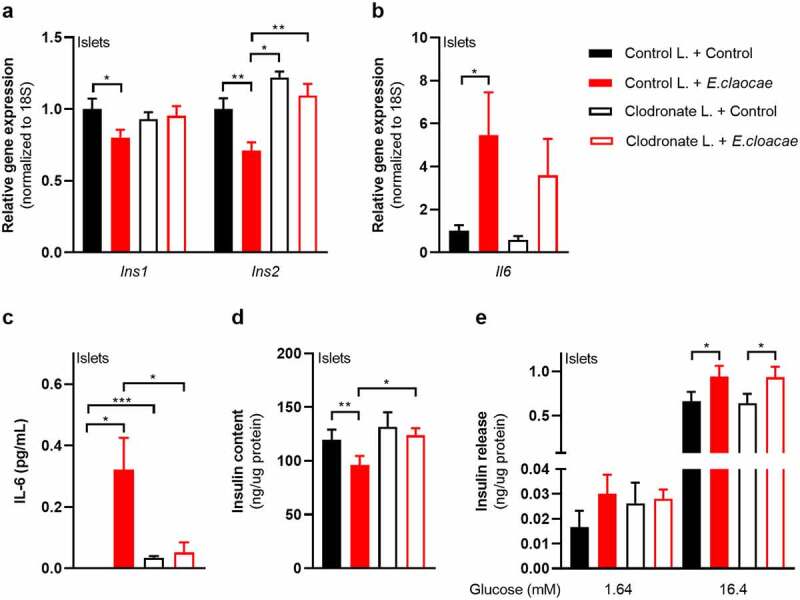
Macrophages mediate beta-cell dysfunction in pancreatic islets.

As TLR5 is barely expressed in beta cells (figure 3f), we concluded that other islet-associated cells in the pancreas, particularly resident islet macrophages, could mediate the observed effects of TLR5 activation. Indeed, macrophages as well as pancreatic islets containing macrophages had higher TLR5 expression than pure beta cells (figure 3f). Macrophages are important for pancreatic islet physiology,^29^ but can induce beta-cell dysfunction when activated towards a pro-inflammatory phenotype.^30^ We thus depleted macrophages from murine pancreatic islets using clodronate-liposomes,^31^ followed by incubation with *E. cloacae* (Figure 4). Macrophage markers were significantly reduced in pancreatic islets after treatment (**Figure S6A**). Reduction of islet macrophages resulted in the maintenance of insulin gene transcription following *E. cloacae* treatment (Figure 4a). Further, expression of IL-6 was slightly reduced (Figure 4b) and its secretion was almost abolished (Figure 4c). Insulin content (Figure 4d) was not affected by *E. cloacae* in islets lacking macrophages. Insulin secretion was slightly increased (Figure 4e). In line with a role for islet resident macrophages, we found that pure beta cells (INS1E clonal cell line) did not show inflammation or beta-cell dysfunction following *E. cloacae* treatment (**Figure S6B-E**). Further, human islet organoids that consist of pure human endocrine cells did not show signs of beta-cell dysfunction upon *E. cloacae* treatment (**Figure S6F-G**). These results collectively indicate that islet-resident macrophages mediate *E. cloacae*-induced islet-cell inflammation and dysfunction.

Macrophage-depleted islets from wild-type C57BL6J mice were treated with heat-inactivated *Enterobacter cloacae* (10^6^ CFUs/mL) for 72h. (a) Macrophage depletion in pancreatic islets protects from *E. Cloacae-* induced reduction in insulin gene expression. (b) *E. cloacae* increases beta-cell inflammation. (c) *E. cloacae* increases beta-cell IL-6 secretion, which is prevented by macrophage depletion in pancreatic islets. (d) *E. cloacae* reduces insulin content, which is prevented by macrophage depletion. (e) *E. cloacae* induces insulin hypersecretion both in WT and in macrophage depleted islets. Data shown are mean ± SEM (three representative experiments per panel, n = 9). Unpaired t-test was used for statistical analysis (a-e). Significance level: *p < .05, **p < .01, ***p < .001, ***p < .001, ****p < .0001. Abbreviations: INS1 and INS2, insulin 1 and 2; IL-6, Interleukin 6.

### Bacterial flagellin induces beta-cell inflammation and dysfunction

Flagellin is the ligand for TLR5,^32^ and therefore we hypothesized that this bacterial component could be the driving force behind the *E. cloacae*-induced phenotype. We isolated flagellin from *E. cloacae* and incubated pancreatic islets with it (**Figure S5C-G**). It induced inflammation and pancreatic beta-cell dysfunction. However, the isolated flagellin showed several impurities. Therefore, we continued with a pure commercial flagellin, which was derived from a very similar bacteria, namely *Salmonella typhimurium* (both from the family Enterobacteriaceae).

**Figure 5.**
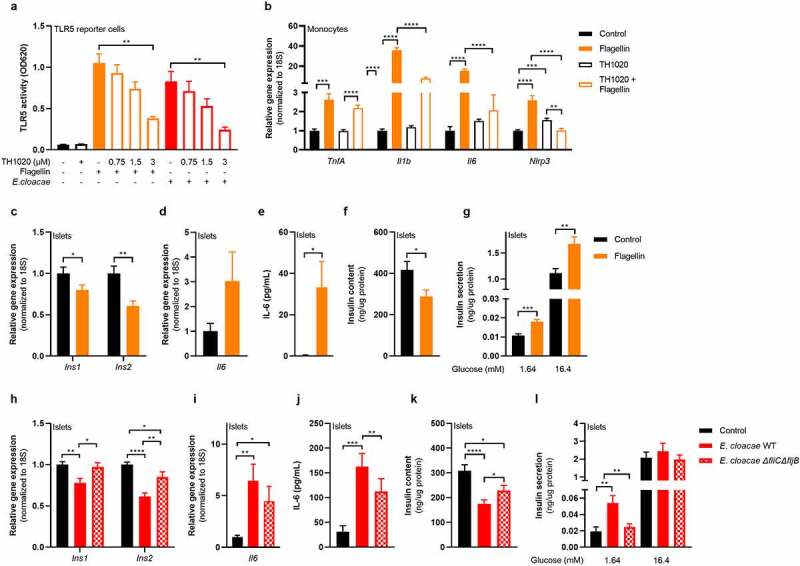
Flagellin induces beta-cell dysfunction in pancreatic islets.

Both flagellin and flagellin-bearing *E. cloacae* activated TLR5 in a human embryonic kidney (HEK) reporter cell line (Figure 5a), which was dose-dependently inhibited by the TLR5 inhibitor TH1020. Furthermore, flagellin induced a pro-inflammatory response in human macrophages, which was reduced when co-incubated with TH1020 (Figure 5b). Residual inflammation might be mediated by NLRC4, which is the second receptor for flagellin.^33^ Additionally, flagellin impaired insulin gene expression (Figure 5c), induced beta-cell inflammation (Figure 5d-e) and reduced insulin content (figure 5f) while increasing insulin release from pancreatic islets (Figure 5g), thus resembling the phenotype induced by *E. cloacae*.

HEK TLR5 reporter cell line (a), human peripheral monocytes (b) and pancreatic islets (c-l) from C57BL6J mice were incubated with flagellin (100 ng/mL) or *E. cloacae* (10^6^ CFUs/mL) for 72h. *E. cloacae* wild-type (a, h-l) or flagellin knockout *E. cloacae* (h-l) was used. (a) Flagellin and *E. cloacae* activate TLR5 in HEK reporter cell line. TLR5 inhibitor TH1020 inhibits receptor activity dose-dependently. (b) Flagellin increases expression of inflammatory genes in monocytes, which is reduced by TLR5 inhibitor TH1020. (c) Flagellin reduces insulin gene expression, (d) increases expression of inflammatory IL-6, (e) increases secreted IL-6, (f) reduces insulin content, (g) induces insulin hypersecretion in islets. (h) Knockout of flagellin in *E. cloacae* partially protects against loss of insulin gene expression,(i) partially protects against inflammatory response, (j) partially protects against enhanced IL-6 release, (k) protects against loss of insulin stores, (l) protects against impaired secretory insulin response. Data shown are mean ± SEM (3 independent experiments, n = 9). Unpaired t-test was used for statistical analysis: *p < .05, **p < .01, ***p < .001, ****p < .0001. Abbreviations: INS1 and INS2, insulin 1 and 2; IL-6, Interleukin 6; FliBC^−/−^, flagellin gene B and C knockout.

To further dissect the role of flagellin in the beta-cell deteriorating effects mediated by *E. cloacae*, we generated an *E. cloacae*-flagellin strain (Δ*fliC*Δ*fljB)* lacking both flagellin genes *fliC* and *fliB.*^34^ *E. cloacae* Δ*fliC*Δ*fljB* reduced insulin expression less than the wild-type (Figure 5h). In addition, expression of inflammatory cytokines and secreted IL-6 were lower in islets exposed to the Δ*fliC*Δ*fljB* strain as compared to islets exposed to wild-type *E. cloacae* (Figure 5i-j). Residual inflammation might come from other bacterial molecules such as LPS. Islets had higher insulin content after incubation with the Δ*fliC*Δ*fljB* strain as compared to wild-type *E. cloacae* (Figure 5k). Finally, the Δ*fliC*Δ*fljB* strain did not induce fasting insulin hypersecretion (Figure 5l). Collectively, these results suggest that flagellin, as part of the flagellum carried by bacteria belonging to Enterobacteriaceae, plays a pivotal role in beta-cell inflammation and beta-cell dysfunction via TLR5 activation on resident islet macrophages.

### Flagellin treatment augments insulin secretion in mice

**Figure 6.**
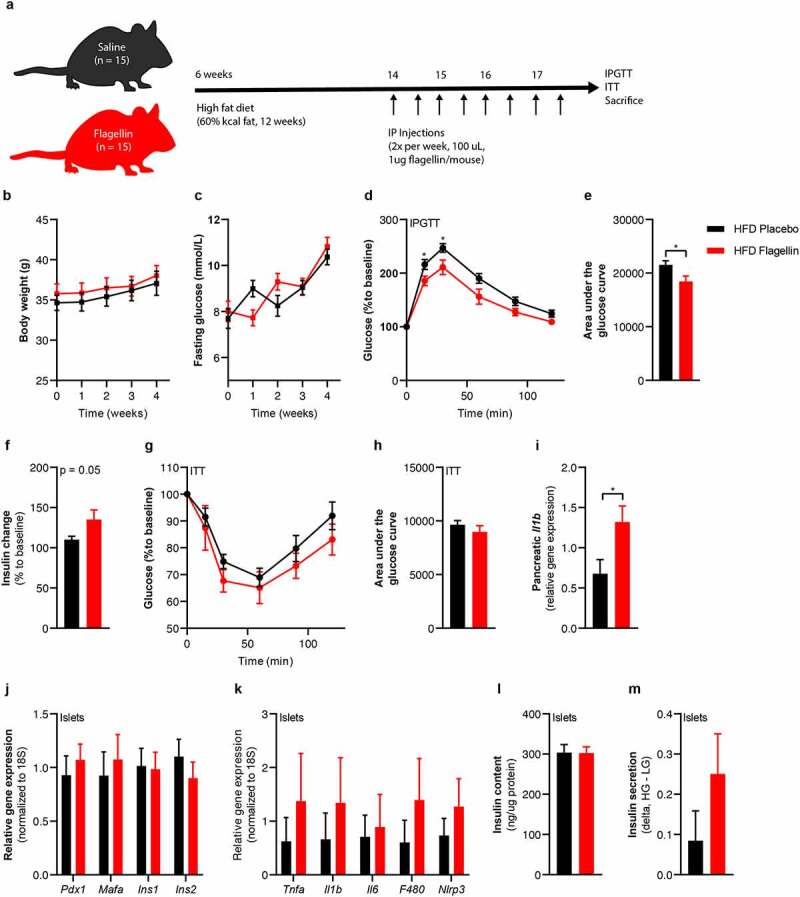
Flagellin injection in mice changes glucose tolerance.

To translate beta-cell dysfunction inducing effects of flagellin to an *in vivo* situation, we injected flagellin intraperitoneally into diet-induced obese (DIO) C57BL6J mice twice weekly for four weeks (Figure 6a). Flagellin injection did not alter body weight or fasting glucose (Figure 6b-c). However, flagellin-treated mice had lower glucose levels during an intraperitoneal glucose tolerance test compared to the placebo group (Figure 6d-e), which was driven by increased insulin secretion (figure 6f) since insulin sensitivity did not differ between groups (Figure 6g-h**, Figure S7**). Similar to the *in vitro* experiments, there was a higher inflammatory tone in the pancreas of flagellin-treated mice as shown by higher *Il1b* expression (Figure 6i) and a trend towards more inflammation in pancreatic islets isolated from flagellin-treated mice (Figure 6k). Although insulin content did not differ between groups, insulin release tended to increase during glucose-stimulated insulin secretion *ex vivo* in islets isolated from the flagellin group (Figure 6j, m). While glucose lowering by increased insulin secretion could be perceived as beneficial, it is important to realize that insulin hypersecretion is related to T2D development over time. As this concerns a process of long duration, prolonged treatment with flagellin is needed to obtain a significant hyperglycemic phenotype. However, our results suggest that flagellin-induced transcriptional and functional alterations in beta-cell function, as observed *in vitro*, can be conceptually replicated *in vivo* in a mouse model.

(a) Six-week-old mice (n = 15) were fed a high fat diet (60% kcal fat) for 12 weeks. In the last 4 weeks of the diet, the mice were injected with either 1 µg flagellin in 100 µL saline or saline alone twice weekly. (b) Flagellin injections do not change the body weight, (c) do not change fasting plasma glucose concentrations. (d, e) Flagellin-injected mice have improved glucose tolerance compared to placebo-treated mice (lower area under the glucose curve). (f) Fold change (15 min to baseline) of plasma insulin is greater in flagellin-treated mice compared to placebo (n = 10). (g, h) Insulin sensitivity is not affected by flagellin injection (0.75 IU/kg). The relative change in glucose to baseline is shown. (i) Flagellin increases pancreatic *Il1b* expression. (j) Flagellin injections do not affect beta-cell gene expression (n = 5), (k) numerically increase markers of beta cell inflammation (n = 5), (l). do not affect insulin content (n = 5) (m), numerically increase insulin release from beta cells (n = 5). Data shown are mean ± SEM. Unpaired t-test (b-m) was used *p < .05, **p < .01, ***p < .001, ****p < .0001. Abbreviations: INS1 and INS2, insulin 1 and 2; NLRP3, NACHT, LRR and PYD domains-containing protein 3; IL-1β, Interleukin 1 beta; IL-6, Interleukin 6; TNF-α, tumor necrosis factor-alpha; ITT, insulin tolerance test.

### Systemic flagellin dissemination relates to beta-cell dysfunction in humans

**Figure 7.**
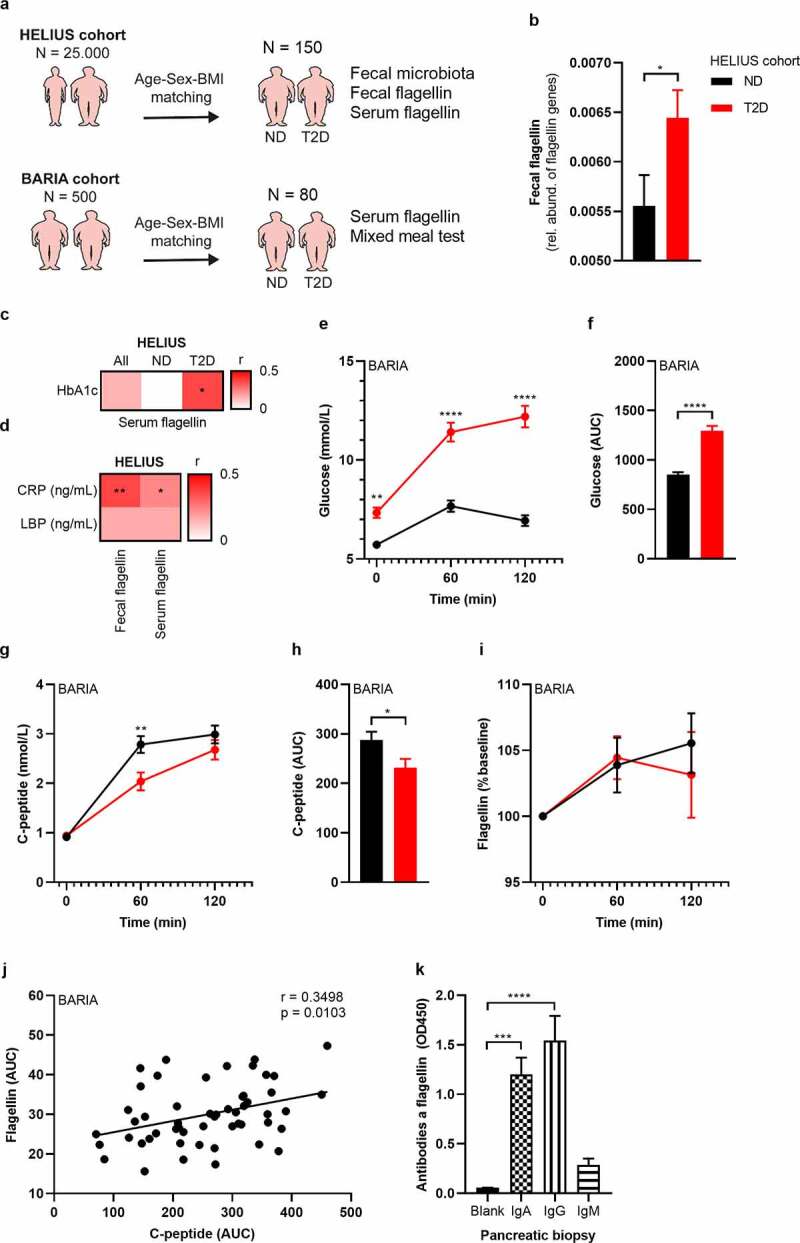
Fecal and serum flagellin is associated with glucose intolerance in humans.

Fecal flagellin has been reported to be increased in obese people compared to lean controls.^20^ Similarly, we observed that obese mice had a higher flagellin load compared to lean mice (**Figure S8**). We predicted fecal flagellin gene abundance in the 150 HELIUS participants by inference from 16S rRNA profiles using PICRUSt (**Table S3**) (Figure 7a). This prediction suggested an increased fecal flagellin abundance in T2D (Figure 7b). Next, we measured bacterial flagellin in the human blood circulation of the HELIUS cohort. While there was no difference between the matched obese groups, we did observe a positive correlation between serum flagellin load and HbA1c in T2D (Figure 7c). Further, the inflammatory marker CRP correlated with fecal flagellin and serum flagellin (Figure 7d). We hypothesized that increased flagellin reaches the circulation following a meal, which was previously shown to drive translocation of endotoxins.^35^ We therefore measured postprandial plasma flagellin, C-peptide and plasma glucose in 80 matched participants of our bariatric surgery cohort^36^ comprising obese normoglycemic and obese T2D people during a mixed-meal test (MMT) (Figure 7a**, Table S4**). C-peptide was chosen as it reflects insulin secretion rates and is not affected by potential differences in clearance by the liver, as is the case for plasma insulin concentrations. The MMT additionally allowed us to study the relationship between meal-induced flagellin and beta-cell response to an MMT. Participants with T2D had hyperglycemia following the MMT (Figure 7e-f) and lower C-peptide concentrations (Figure 7g-h) compared to normoglycemic humans with obesity. In both groups, flagellin increased during the MMT (Figure 7i). Postprandial area under the curve (AUC) for flagellin correlated with AUC C-peptide (Figure 7j), highlighting the link between bacterial flagellin and beta-cell insulin secretion in human beta-cell physiology. Interestingly, this correlation is mostly driven by individuals without diabetes (ND r= 0.6, p= .001; T2D r= 0.069, p= .736), potentially due to the presence of beta-cell failure in T2D.

(a) 150 people were randomly selected from the HELIUS cohort.^14^ Participants with T2D were matched with normoglycemic controls according to age, sex and BMI. In addition, 80 participants were selected from the bariatric surgery cohort (BARIA).^36^ (b) Fecal flagellin genes are increased in T2D, as inferred from 16S rRNA gene profiles (HELIUS). (c) Correlation matrix between serum flagellin and glucose marker HbA1c (HELIUS). (d) Correlation matrix of CRP and LBP with fecal flagellin and serum flagellin (HELIUS). (e-f) Plasma glucose concentrations during a mixed meal test (MMT, BARIA). (g, h) Plasma C-peptide concentrations during the MMT (BARIA). (i) Serum flagellin increased during the MMT (BARIA). (j) A positive correlation between serum flagellin AUC and plasma C-peptide AUC during the MMT (BARIA) exists. (k) Antibodies against bacterial flagellin were measured in homogenized human pancreatic biopsies via ELISA. Data shown are mean ± SEM. Unpaired t-test (b-h) and Spearman correlation (i) were used: *p < .05, **p < .01, ***p < .001, ****p < .0001. Abbreviations: HELIUS, Healthy Life in an Urban Setting; AUC, Area under the curve; MMT, mixed meal test; Ig, immunoglobulin; T2D, type 2 diabetes; BMI, body mass index; ND, no diabetes; CRP, c-reactive peptide; LBP, LPS binding protein.

Finally, to support the hypothesis that systemically disseminated flagellin causes the observed beta-cell phenotype, we collected pancreatic biopsies from people with T2D undergoing pancreatic surgery for benign lesion. We collected five biopsies of the head of the pancreas. Recent antibiotics use (<3 months) was an exclusion criterion (**Table S5**). While it was technically not possible to measure flagellin in these biopsies due to interference of the pancreatic enzymes with the flagellin assay, we observe the presence of antibodies against flagellin, supporting the notion that the well-vascularized pancreatic islets are exposed to flagellin (Figure 7k). A previous study indicated that a functional immune response is essential to control flagellin expressing bacteria,^37^ which seems to be reduced in obese humans.^20^

## Discussion

In this study, we reveal a novel pathway by which flagellin, a structural component of bacteria residing in the gut, systemically disseminates following food ingestion. In pancreatic islets that are abundantly vascularized,^38^ we propose that flagellin activates the innate immune system and induces an inflammatory response following binding to TLR5 receptors expressed by resident islet macrophages. This leads to beta-cell dysfunction, characterized by impaired insulin gene expression, impaired insulin processing, insulin hypersecretion and reduced insulin content. This study provides a new insight into the link between gut microbiota composition and T2D.

Beta-cell dysfunction is the key abnormality that leads to the development of hyperglycemia and T2D. Beta-cell dysfunction is characterized by inappropriate insulin secretion, which can either be excessive or insufficient, upon exposure to glucose or other nutrients.^39^ The role of hyperinsulinemia in T2D development has received ample attention. While initially reported to be a response against obesity-related insulin resistance, research has indicated that increased insulin secretion can develop in the absence of insulin resistance implying primary beta-cell pathology.^40^

Induction of hyperinsulinemia has been shown to promote obesity,^4^ while prevention of hyperinsulinemia by pancreas-specific genetic knockout of insulin expression prevented obesity, improved insulin sensitivity and did not result in overt hyperglycemia.^4^ With respect to the pancreatic islets, a chronic demand on beta cells to produce insulin is detrimental. As such, a prolonged increase in insulin secretory rates have been related to endoplasmic reticulum (ER) stress, depletion of intracellular insulin stores and beta-cell apoptosis.^41^ Pancreatic islets from individuals with T2D have lower insulin content compared to healthy controls.^42^ In mice, hyperglycemia leads to insulin content loss.^43^ Reversibly, strategies that induce beta-cell rest are linked to improved beta-cell function over time.^44^

Current evidence relates overnutrition of carbohydrates and non-esterified fatty acids to beta-cell dysfunction.^45^ Another factor concerns a low-grade inflammatory response^46^ and ER stress.^47^ Beta-cell inflammation is a known hallmark of islets in people with T2D,^48^ and a central role in this regard has been proposed for islet macrophages.^6^ Macrophages are essential for normal beta-cell function and physiology,^29^ however, macrophages with a pro-inflammatory phenotype have been linked to beta-cell dysfunction.^31^ Triggers for activation of pro-inflammatory macrophages are uncertain but may involve hyperglycemia,^49^ dyslipidemia^50^ and human islet amyloid polypeptide (hIAPP).^51^ Here, we show that *E. cloacae* and flagellin induce inflammation in macrophages. Depleting pancreatic islets from macrophages reduced the deleterious effect on beta cells; however, we need to acknowledge that we only tested the compounds on monocyte-derived macrophages and not isolated islet resident macrophages.

In the present study we show an infectious stimulus triggering inflammation and beta-cell dysfunction: flagellin derived from intestinal microbiota. While Gram-negative bacteria are known to produce canonical flagellin, which is studied here, several other intestinal Firmicutes species are motile and have been described to contain flagella, including several *Roseburia, Clostridium*, and *Lactobacillus* spp.^52^ However, the flagellins of these latter, Gram-positive bacteria have not been well characterized and some are glycosylated resulting in attenuated TLR5 signaling efficiency.^53^

A first link towards flagellin came from the observation that the fecal abundance of the family of Enterobacteriaceae, specifically *E. cloacae*, was increased in people with T2D and that the fecal abundance of Enterobacteriaceae and *E. cloacae* correlated with glucose intolerance in humans. Administration of *E. cloacae* by oral gavage to mice fed a high-fat diet has previously been shown to induce glucose intolerance.^18^ We used one strain of *E. cloacae* that affected glucose tolerance in mice and induced beta-cell dysfunction. We speculate that the whole *E. cloacae* complex and several other pro-inflammatory bacteria belonging to the family of Enterobacteriaceae have similar effects, however that still needs to be tested. A proposed mechanism by which gut microbiota may influence host metabolism is by escaping immune control and translocating to extra-intestinal tissues.^23^ While this has been shown for adipose tissue,^54^ translocation of intestinal bacteria into the pancreas was also suggested to trigger the influx of immune cells and islet inflammation.^55^ Here, we used *E. cloacae* as a proof-of-concept bacterium. Several other bacteria belonging to the group of Enterobacteriaceae, such as *E. coli*, have similar effects on the human metabolism. Further, we used heat-inactivated bacteria that might induce a different immune response than viable bacteria

In patients undergoing pancreatoduodenectomy, pancreatic fluid contained bacterial DNA, with a similar composition, density and diversity as bile and jejunal fluid,^56^ suggesting direct translocation from the small intestine into pancreatic juice. Others also suggest a bacteriome^57^ and mycobiome^58^ in pancreatic tissue of cancer patients. In line with these data, we observe a correlation between systemic antibodies against *E. cloacae* and hyperglycemia, which may suggest translocation of at least parts of this bacteria to extraintestinal sites.^59^ Nevertheless, translocation of whole bacteria remains rather controversial^9^ since there are major challenges related to the sequencing of small amounts of bacterial DNA in extraintestinal tissues.^60^

Heat-inactivated *E. cloacae* induced a detrimental beta-cell phenotype with insulin hypersecretion, induction of ER stress markers (elevated PI/I ratio), inflammation and reduced beta-cell insulin content. TLR4 can detect bacterial LPS and is involved in beta-cell dysfunction.^21^ Surprisingly, TLR4 knockout did not protect from bacteria-induced insulin expression loss, but did reduce the expression and secretion of IL-6. IL-6 is involved in glucose tolerance and beta-cell dysfunction, but its function is far from understood.^61^ On the other hand, deletion of TLR5, of which flagellin is the dominant ligand, protected against the insulin expression loss after incubation with flagellum-bearing *E. cloacae*. We speculate that an imbalance of TLR5 signaling drives metabolic diseases.

Flagellum is a virulence factor that enables bacteria to move within the intestine and even adhere to the intestinal wall, a process called encroachment.^20^ Flagellin is expressed by a variety of bacteria. Here, we used a commercially available pure flagellin from *Salmonella typhimurium* to avoid impurities from other bacterial components such as LPS. Both bacteria, *S. typhimurium* and *E. cloacae*, belong to the family Enterobacteriaceae. We see similar effects for both flagellin types. Flagellin fully reproduced the beta-cell phenotype of *E. cloacae*. A causal role for flagellin was observed in *E. cloacae* with flagellin knockout, where the effects on inflammation, hypersecretion and reduced insulin content were strongly diminished. Strengthening the role of flagellin, mice that were injected with flagellin exhibited a similar beta-cell phenotype. However, we cannot deduce whether this effect was directly on beta cells or a secondary response on other cells.

Flagellin, like the widely studied LPS, may translocate after food ingestion. Importantly, flagellin is able to pass the epithelial barrier^62^ and is a potent stimulus of the mucosal immune response.^37^ Plasma flagellin levels also positively correlated with HbA1c in our cohort, while the meal-related flagellin increment was associated with higher C-peptide release in obese humans with or without diabetes. Further, we found positive correlations between systemic inflammatory markers CRP and LPB with BMI, glucose values and antibodies against *E. cloacae*, suggesting that this bacteria induces inflammation in the host.

We acknowledge a number of limitations of this study. First, although the concept of translocation of flagellin to the systemic circulation seems to be plausible to explain beta-cell inflammation,^55^ we can only speculate if bacterial flagellin is transported via the blood circulation toward the pancreas. More sensitive methods are necessary to quantify small amounts of bacterial components such as flagellin in extra-intestinal tissues.^20^ Administration of single bacteria, for example flagellin bearing *E. cloacae* as well as our knockout strain, in mice would help to answer whether flagellin induces glucose intolerance. However, single strains do not engraft in the SPF murine microbiome and likely do not show an effect.^18^

Second, we provide evidence that people with T2Ds have a higher fecal and circulating flagellin load compared to normoglycemic individuals, with flagellin loads correlating with hyperglycemia. However, such a correlation does not show causation. With our data, we cannot decipher the role of flagellin in prediabetes and overt T2D. We can propose that flagellin induces insulin hypersecretion in early stages of the disease and longitudinal studies need to be done to show that this results in hypoinsulinemia and hyperglycemia over time. Insulin hypersecretion remains a controversial topic. Here we show that flagellin injection increases insulin secretion, beta-cell inflammation, but also reduces glucose levels. Therefore, resembling pre-diabetic states. However, achieving a full hyperglycemia in mice is only possible with genetic modulation and chemical treatment. Following these mice for a longer period would potentially show a more severe phenotype; however, not tested here. Together, we present a novel pathway linking bacterial flagellin from the Gram-negative *E. cloacae* in a TLR5-macrophage-dependent manner to beta-cell inflammation and beta-cell dysfunction, suggesting a new mechanism linking gut microbiota and T2D prevalence and opening up potential avenues for novel therapies.

## Methods

### Participants

For the current study, we included all 803 people of Dutch descent with available data on the gut microbiome from the HELIUS cohort in Amsterdam, the Netherlands.^14^ For details, regarding the HELIUS study (recruitment, data collection) in general, and regarding this selection in particular, see Deschasaux et al.^15^ For serum analysis on LPS and flagellin, 150 participants were randomly selected and diabetic participants (n = 100) were age-BMI-sex matched to healthy non-diabetic controls (n = 50). Diabetic participants were selected according to one of the following criteria (at least one): self-reported diagnosis of T2D, use of antidiabetic medication, fasting blood glucose >7.0 mmol/L and HbA1c >48 mmol/mol. All participants did not use antibiotics for the last 3 months.

The HELIUS data are owned by the Amsterdam UMC, location AMC in Amsterdam, The Netherlands. Any researcher can request the data by submitting a proposal to the HELIUS Executive Board. The HELIUS Executive Board will check proposals for compatibility with the general objectives, ethical approvals and informed consent forms of the HELIUS study.

To validate our results in the HELIUS cohort, we included 40 T2D participants and 40 non-diabetic, age, sex and BMI matched controls of the BARIA cohort. For details, see Van Olden et al.^36^ The BARIA study aims to assess how microbiota and their metabolites affect transcription in key tissues and clinical outcome in obese subjects and how baseline anthropometric and metabolic characteristics determine weight loss and glucose homeostasis after bariatric surgery.

Human pancreatic islets were obtained from Prodo Labs (US). Donor characteristics can be found in **Table S7**.

### Bacteria

*Enterobacter cloacae* NCDC 279–56 and *Escherichia coli* K12 were purchased from German Collection of Microorganisms and Cell Cultures (DSMZ, Germany). They were cultured in Luria broth base (Invitrogen, US) and on LB Agar (Invitrogen, US) at 37°C, overnight, before being used for experiments. *Faecalibacterium prausnitzii* A2-165 was cultured in YCFA media anaerobically and *Bacteroides ovatus* 3_8_47FAA in YZFAA media anaerobically. Both were a generous gift from Willem de Vos (Wageningen).

### Animals

Ten-week-old male C57BL/6 J mice were purchased from Charles River (France) and maintained under specific pathogen-free conditions in the S-building of the Amsterdam UMC, location AMC. TLR2 KO, TLR4 KO, TLR5 KO and C57BL/6 J DIO mice were purchased from Jackson Laboratory (JAX); control animals on C57BL/6 J background were used from JAX facilities instead of Charles River. All animals were socially housed, under a 12 h light/dark cycle until 12–14 weeks and sacrificed for pancreatic islet isolation. Only male mice were included in this study.

For flagellin injection, male obese C57BL6/J mice were ordered from JAX at 13 weeks (n = 15) and acclimatized for 1 week. Power was calculated according to insulin secretion after LPS injection^63^ with nQuery software (version 8.5.1). Mice were maintained on a high fat diet (60% kcal fat, ResearchDiets). Three to four mice were housed per cage. At week 14, mice were intraperitoneal injected twice weekly with 1 µg flagellin (Invivogen) in 100 uL saline for 4 weeks. Cages were randomly assigned to the treatments. Control mice were injected with100 µL saline. Body weight was measured before and during injections. Animals were fasted for 4h to measure fasting glucose. A intraperitoneal glucose tolerance (IPGTT) test was performed after the last injection (1.5 g/kg glucose in saline). Insulin was measured from the tail vein at baseline and 15 minutes in a subset of animals (n = 10) due to practical limitations. Insulin tolerance test was performed with 0.75 IU/kg (NovoRapid insulin). Glucose was measured in the tail vein blood every 15–30 minutes during 2 hours. Mice were killed with the aid of isoflurane and cardiac puncture. Organs were snap frozen. From a subset of mice (n = 5) due to practical limitations, pancreatic islets were isolated as described above, rested for 3 h in full media and RNA was harvested as well as GSIS was performed. Animal work was performed in accordance with the Central Commission for Animal Experiments (CCD, The Netherlands).

### Heat-inactivation of bacteria

The optical density of the bacterial culture was measured at 600 nm (OD600) and diluted to 1E9 colony forming units (CFUs) per mL. Bacteria were centrifuged at 8000 xg for 5 minutes and resuspended in 1 mL sterile phosphate buffered saline (PBS). All bacteria were heat-inactivated at 70°C for 30 min and stored at −80°C in small aliquots for further use.

### Pancreatic surgery

Individuals who are scheduled for pancreatic surgery (e.g., pylorus-preserving pancreatoduodenectomy or Whipple’s procedure), because of pancreatic carcinoma, were asked to donate healthy tissue surrounding the tumor. Tissue was harvested under surgical conditions, snap frozen in liquid nitrogen and stored at −80°C until further analysis.

### Pancreatic islet isolation

Mice were anaesthetized with 2.5 mg pentobarbital (diluted in sterile saline) per mouse and sacrificed via cervical dislocation. After clamping the *Ampulla of Vatar*, the pancreas was injected intraductally with approximately 3 mL of collagenase XI (1000 U/ml) in HBSS (without calcium chloride) and placed in 50 mL tubes with an additional 2 mL of collagenase solution. The pancreas was incubated at 37°C for 13 minutes followed by gentle shaking to obtain a homogeneously dispersed pancreas. Digestion was stopped with cold HBSS supplemented with 1 mM CaCl_2_. Islets were washed two times in cold HBSS with CaCl2 by centrifuging 185 xg for 30 seconds. Next, islets were filtered through a 70 μM prewetted cell strainer. After flushing two times with 10 mL of HBSS with CaCl_2_, the strainer was turned upside-down over a Petri dish and rinsed with 16 mL of islet media (RPMI 1640 with GlutaMAX^TM^ 1x, 10% FBS and P/S 1x) to collect the islets into the dish. Islets were handpicked under the Nikon SMZ800 microscope into a fresh Petri dish with islet media. Islets were rested overnight to recover from isolation procedure. Fresh pancreatic islets were incubated with 10^6^ CFUs of heat-inactivated bacteria for 72 h at 37°C in full RPMI (1x Glutamax, 10% FBS, Pen/Strep). Islets were picked out of the media and used for GSIS (10 islets per replicate, in triplicates) or RNA isolation (50 islets per replicate, in triplicate). Media was saved at −80°C.

### Plasmid construction

Overlap extension PCR^64^ was used to generate pRE118-pheS-ΔfliC and pRE118-pheS-*ΔfljB* constructs (pRE118-pheS was a gift from Christopher Hayes of UC Santa Barbara). For pRE118-pheS-*ΔfliC* construct, two PCR fragments were amplified using *E. cloacae* genomic DNA as the template. Primer pairs used to amplify the PCR fragments are ecFliC-P1 (5’-GATGATGGTGATGGTACGCGTGGTACCGGTAGTCGCT-3’) plus ecFliC-P2 (5’-GGTTTCTAGGGTCGGTGCCTTAACACTCA-3’), and ecFliC-P3 (5’- CACCGACCCTAGAAACCCTGTCTCTGCTGCGTTAA-3’) plus ecFliC-P4 (5’-GACAGTGAGCTCGCATCGTTAACGCGTCTTCACCAA-3’), respectively. This results in a 789-bp fragment containing the upstream of *fliC* and a 750-bp fragment containing the downstream of the *fliC*, respectively. These two PCR fragments were then mixed and used as the template for a secondary PCR (with primer pairs ecFliC-P1 containing a KpnI restriction enzyme site and ecFliC-P4 containing a SacI restriction enzyme site). The 16-bp overlapping sequence in primers ecFliC-P2 and ecFliC-P3 allows the amplification of a 1,539-bp PCR product. This PCR product was digested with KpnI and SacI, and directly cloned into the *E. cloacae* suicide vector pRE118-pheS (Kanr).

The pRE118-pheS-Δ*fliC* construct was generated the same as above. Primer pairs used to amplify the PCR fragments are FljB-P1 (5’-GCACGTCTAGAGTGACCTTTATCGTCATCTCACCGT-3’) plus FljB-P2 (5’-GTACCCAGCTGAGTCTGGGATTTGTTCAGGTTGTT-3’), and FljB-P3 (5’- AGACTCAGCTGGGTACTGCTGCGTTAATCTGCGTTA-3’) plus FljB-P4 (5’-GACAGTGAGCTCGTACAGCTATTCGCTGCATAACGA-3’), respectively. This results in a 955-bp fragment containing the upstream of *fljB* and a 950-bp fragment containing the downstream of the *fljB*, respectively. These two PCR fragments were then mixed and used as the template for a secondary PCR (with primer pairs FljB-P1 containing a XbaI restriction enzyme site and FljB-P4 containing a SacI restriction enzyme site). The 16-bp overlapping sequence (underlined) in primers FljB-P2 and FljB-P3 allows the amplification of a 1,905-bp PCR product. This PCR product was digested with XbaI and SacI, and directly cloned into the E. cloacae suicide vector pRE118-pheS.

### Generation of *E. cloacae* mutant strains

pRE118-pheS-*ΔfliC* and pRE118-pheS-*ΔfljB* constructs were transformed into *E. coli* MFD(λ pir). E. coli MFD(λ pir) carrying these constructs and WT *E. cloacae* were grown overnight in LB, and then mixed at a ratio of 4:1 (donor vs recipient strains). To make fljB fliC, E. coli MFD(λ pir) carrying pRE118-pheS-*ΔfljB* and *ΔfliC* were grown overnight in LB, and then mixed at a ratio of 4:1. Fifty microliters of the mixture was spotted onto LB agar plate containing diaminopimelic acid (DAP, 0.3 mM) and incubated at 37°C overnight. This was followed by scraping the cell mixtures in PBS and plating onto LB agar containing streptomycin (100 μg/ml) and kanamycin (50 μg/ml). The resulting single-crossover mutants were grown statically in LB at 37°C overnight, and further counter selected on M9 minimal medium agar plates containing 0.4% (w/v) glucose and 0.1% (w/v) p-chlor-ophenylalanine.^65^ Kanamycin sensitive colonies were screened by colony PCR. The ΔfliC deletion mutant was confirmed by PCR with primers ecFliC-check-F (5’-GCGTTTCTGATGGCGTTCTGAA-3’) and ecFliC-check-R (5’-GCTCGAACTTGTTCATCCCGATT-3’). The predicted size of WT and mutant bands is 1201-bp and 362-bp, respectively. The *ΔfljB* deletion mutant was confirmed by PCR with primers FljB-check-F (5’-GCAGAACAACCTGAACAAATCCCA-3’) and FljB-check-R (5’-GACACGTTTACGCCGGTTCACTAT-3’). The predicted size of WT and mutant bands is 1811-bp and 387-bp, respectively.

### Confirming mutants with a swimming motility assay

WT and mutant E. cloacae strains (*ΔfljB, ΔfliC, ΔfljBΔfliC*) were grown statically in 2 μl of LB at 30°C for 18 h. Two microliters of these cultures were spotted onto semi-solid nutrient broth (BD) agar plates containing 0.3% agar. After incubating the plates at 37°C for 4 h, pictures showing the swimming motility were taken (**Figure S9**).

### Glucose stimulated insulin secretion

Pancreatic islets or β-cell lines were washed in a 12 well plate 2x with 500 uL low glucose Krebs-Ringer buffer (KRB; 132 mM NaCl, 5 mM KCl, 1 mM KH_2_PO_4_, 1 mM MgSO_4_, 2.5 mM CaCl_2_, 5 mM NaHCO_3_, 10 mM HEPES, 0.25% BSA, 1.64 mM glucose) and starved in 500 uL low glucose KRB for 1 h. Islets were split into 10 islets per well in a 12 well plate (triplicates) and incubated for 1 hours in 500 uL low glucose KRB. The same islets were transferred into 500 uL high glucose KRB (16.4 mM) for 1 h. Finally, islets were washed 2x with 1 mL PBS and lysed with 150 uL RIPA buffer. Islet lysate was spun at 14.000 xg for 10 min at 4°C, and the supernatant was stored at −20°C until further use.

### DNA isolation

Genomic DNA was isolated from overnight bacteria cultures with QIAamp® DNA Blood Mini Kit (Qiagen, Germany) according to manufacturer's instructions.

Fecal DNA was extracted from 150 mg fecal material and the sorted fractions using a repeated bead beating protocol (method 5).^66^ DNA was purified using Maxwell RSC Whole Blood DNA Kit. 16S rRNA gene amplicons were generated as described below.

### RNA isolation and cDNA synthesis

RNA was isolated with TriPure^TM^ isolation reagent (Roche). Cells were separated from the culture media, and 300 uL TriPure^TM^ was added. After lysis, 60 uL chloroform was added, the mixture was vigorously shaken for 15 seconds and incubated for 3 minutes at room temperature. Next, samples were spun for 15 minutes at 12.000 xg (4°C) and the aqueous phase was mixed with 190 uL isopropanol with 0.44 uL GlycoBlue^TM^. After an overnight incubation at −20°C, samples were spun at 12.000 xg for 10 minutes (4°C) and the pellet was 2x washed with 1 mL of 75% ethanol (7.500 xg, 5 minutes, 4°C). Next, the pellet was dried at room temperature for 10 minutes, 18 uL RNase free H2O was added and incubated at 56°C for 10 minutes. RNA concentration was measured with Nanodrop. cDNA synthesis was performed with SensiFAST^TM^ cDNA Synthesis Kit according to manufacturer's instructions.

### PCRs

Gene expression was measured *via* real-time quantitative PCR (RT-qPCR) with the aid of PCR machine (BioRad, US). SensiFAST^TM^ SYBR® No-ROX Kit was used according to manufacturer's instructions. For each well, 7.5 ng cDNA and 1 μM primer mix were used in a 10 uL PCR mix. For primers, see **Table S6**. Temperatures are used as following, if not stated differently: 95°C for 10 minutes, 40 cycles of 95°C for 15 seconds and 60°C for 30 seconds with a plate reading, followed by a melt curve with increment of 0.5°C every 5 seconds starting from 65°C to 95°C. TLR5 expression was measured in cDNA from pure beta cells (rat INS1E cells), murine pancreatic islets and human monocytes. Three different sets of primers were used for each species and normalized to 18S reference gene (see primer list, **Table S6**).

Fecal bacteria were measured via quantitative PCR with the aid of PCR machine (BioRad, US). SensiFAST^TM^ SYBR® No-ROX Kit was used according to manufacturer's instructions. For each well, 10 ng genomic DNA and 300 nM primer mix were used in a 10 uL PCR mix. For primers, see **Table S6**. For the total bacteria in feces, EUBAC primers and temperature settings were used as stated.^67^ For Enterobacteriaceae detection, En-lsu3 was used as described.^68^ Primers for *Enterobacter cloacae* was designed for the V3V4 regions. Temperatures as described above were used. Standard amplicons were made with genomic DNA from *E. coli* or *E. cloacae* and *Taq* DNA Polymerase (Qiagen, Germany) according to manufacturer’s instructions. Amplicons were cleaned with QIAquick PCR purification Kit (Qiagen, Germany). Copy numbers were calculated according to the standard curve.

### Cell lines

HEK-Blue^TM^ hTLR5 cells were used according to manufacturer's instructions. For LPS and flagellin detection in the blood circulation, 20 uL serum was used per well (96 well plate) and mixed with 180 uL of 1.4x1E5 cells/mL in detection media. Cells were incubated for 16 hours and the supernatant was read at OD655. INS-1E cells were cultured in RPMI 1640 media (5% FBS, 1x P/S, 1x HEPES, 50 μM β-mercaptoethenol, 1x sodium pyruvate) and passaged with 0.25% Trypsin-EDTA. Cells were seeded in a 12 well plate at 75.000 cells/mL, rested overnight and incubated with heat-inactivated bacteria for 72 hours.

### Antibody analysis

Bacteria were grown overnight, and the optical density was measured at OD600. Bacteria were diluted to have 1E9 CFUs/mL and washed with 1 mL sterile PBS (8000 xg, 5 minutes, 4°C). Bacteria were sonicated on ice at 30% amplitude for 20 × 30 seconds cycles with 60 seconds intervals. Nunc^TM^ MicroWell^TM^ 96-well microtiter plates (ThermoScientific^TM^, US) were coated with 200.000 sonicated bacteria per well (100 uL) overnight at 4°C. Plates were washed 3x with 300 uL per well of PBS and blocked with 150 uL PBS with 1% BSA for 2 hours at room temperature. Plates were washed again with PBS, and 100 uL of 250x diluted serum samples (PBS/BSA) was added and incubated for 4 hours at room temperature. Plates were washed 3x with PBS with 0.05% Tween 20 and 100 uL of secondary antibody (2000x diluted HRP anti-human IgG; 50.000x diluted HRP anti-human IgM; 20.000x diluted HRP anti-human IgA; in PBS/Tween 20) was added for 2 hours at room temperature. Plates were washed with PBS again, and 100 uL of TMB was added for 15 minutes. The reaction was stopped with 50 uL of 0.5 M HCl and read at OD450.

Pancreatic biopsies were 10x diluted according to tissue weight and homogenized in ultrapure water (Invitrogen, US) with the aid of a sterile metal bead. The homogenate was spun Nunc^TM^ MicroWell^TM^ 96-well microtiter plates (ThermoScientific^TM^, US) were coated overnight (4°C) with 100 ng per well of flagellin from *Salmonella typhimurium* (Invivogen, US). The plates was washed 3x with 300 uL PBS/Tween 20. Afterward, 100 uL of homogenized pancreas was added and incubated for 1 h at 37°C. The plates was washed 3x with 300 uL PBS/Tween 20. Secondary antibodies and TMB were added as described above.

### ELISA

Insulin was measured in low glucose KRB, high glucose KRB and cell lysate from GSIS experiments with ALPCO mouse ultrasensitive insulin ELISA according to manufacturer's instructions. Concentrations were normalized to total protein content measured via QuantiPro^TM^ BCA Assay Kit. IL-6 concentrations were measured in cell supernatants *via* ELISA MAX^TM^ Deluxe Set Mouse IL-6 and IL-6 human uncoated ELISA kit according to manufacturer's instructions. Proinsulin was measured with Mercodia Rat/Mouse Proinsulin ELISA (Schweden). Lysates or glucose media from GSIS experiments were used. Ratios were calculated by dividing proinsulin with insulin for each of the nine replicates. CRP and LBP were measured in serum according to the manufacturer’s instructions (HycultBiotech, NL).

### *Isolation of flagellin from* E. cloacae

*E. cloacae* was grown overnight in LB media. Bacteria were pelleted at 4000xg for 5 min. Pellet was washed with PBS. The pellet was resuspended in PBS and vigorously shaken for 2 min at 6 m/s (FastPrep). Bacteria were pelleted, and the supernatant was spun at 100.000 xg for 1 h at 4°C. The pellet containing flagellin was resuspended in PBS and heated at 70°C for 20 min to depolymerize the filaments. Protein concentrations were measured with BCA according to the protocol (ThermoFisher, US). TLR5 activity was verified on HEK TLR5 reporter cell line (Invivogen, US).

### Monocyte isolation

PBMCs were isolated with Lymphoprep (GE Healthcare) and CD14 MACS beads (Miltenyi) according to manufacturer's instructions.

### Macrophage depletion

Pancreatic islet macrophages were depleted with Clodronate-liposome. Islets were isolated and rested for 3 hours. Islets were picked in a small petri dish (40–70 islets per dish) and treated with either clodronate or control liposome for 48 h (1 in 5 diluted in islet media). Islets were washed 3x with 2 mL complete media and picked in fresh media.

### Library preparation and sequencing

Library preparation and sequencing was performed at the Wallenberg Laboratory (Sahlgrenska University of Gothenburg, Sweden). Fecal microbiome composition was profiled by sequencing the V4 region of the 16S rRNA gene on an Illumina MiSeq instrument (Illumina RTA v1.17.28; MCS v2.5) with 515 F and 806 R primers designed for dual indexing^69^ and the V2 Illumina kit (2x250 bp paired-end reads). 16S rRNA genes from each sample were amplified in duplicate reactions in volumes of 25 µL containing 1x Five Prime Hot Master Mix (5 PRIME GmbH), 200 nM of each primer, 0.4 mg/ml BSA, 5% DMSO and 20 ng of genomic DNA. PCR was carried out under the following conditions: initial denaturation for 47 min at 94°C, followed by 25 cycles of denaturation for 45 s at 94°C, annealing for 60 s at 52°C and elongation for 90 s at 72°C and a final elongation step for 10 min at 72°C. Duplicates were combined, purified with the NucleoSpin Gel and PCR Clean-up kit (Macherey-Nagel) and quantified using the Quant-iT PicoGreen dsDNA kit (Invitrogen). Purified PCR products were diluted to 10 ng/μL and pooled in equal amounts. The pooled amplicons were purified again using Ampure magnetic purification beads (Agencourt) to remove short amplification products; for negative controls, see Deschasaux, Bouter, Prodan, Levin, Groen, Herrema, Tremaroli, Bakker, Attaye, Pinto-Sietsma, van Raalte, Snijder, Nicolaou, Peters, Zwinderman, Backhed and Nieuwdorp.^15^ Libraries for sequencing were prepared by mixing the pooled amplicons with PhiX control DNA purchased from Illumina. The input DNA had a concentration of 3 pM and contained 15% PhiX and resulted in the generation of about 700 K clusters/mm2 and an overall percentage of bases with quality score higher than 30 (Q30) higher than 70%.

### Bioinformatic pipeline

USEARCH (v11.0.667_i86linux64) was used to process the raw sequencing reads. For paired-end merging, we used 30 max. allowed differences in the overlapping region (“maxdiffs”) for the merging step (using the “fastq_mergepairs” command) and max. 1 expected errors (“fastq_maxee”) as a quality filter threshold (using the “fastq_filter” command). Expected error-based read quality filtering is described in detail in Edgar et al. 2015. After merging paired-end reads and quality filtering, remaining contigs were dereplicated and unique sequences were denoised using the UNOISE3 algorithm in order to obtain Amplicon Sequence Variants (ASVs). All merged reads were subsequently mapped against the resulting ASVs to produce an ASV table. ASVs not matching expected amplicon length were filtered out (i.e. ASV sequences longer than 260 bp or shorter than 250 bp). Taxonomy was assigned with the ‘assignTaxonomy’ function from the ‘dada2’ R package (v 1.12.1) and the SILVA (v. 132) reference database. ASV sequences were then aligned using MAFFT (v.7.427) using the auto settings. A phylogenetic tree was constructed from the resulting multiple sequence alignment with FastTree (v.2.1.11 Double Precision) using a generalized time-reversible model (‘-gtr’). The AVS table, taxonomy and tree were integrated using the ‘phyloseq’ R package (v.1.28.0). The ASV table was rarefied to 14,932 counts per sample with vegan v2.5–6. Of 6056 sequenced samples, 24 had insufficient counts (<5000 counts per sample) and were excluded at the rarefaction stage. The final dataset thus contained 6032 samples and 22,532 ASVs. Functional composition was inferred using PICRUSt2 (2.2.0b).

### Cell viability

Cell viability of was measured with CellTiter-Glo® Luminescent Cell Viability Assay (Promega) according to manufacturer’s instructions. Ten size-matched islets were used per replicate with five replicates per experiment. Luminescence was read with Promega GLOMAX^TM^ multi detection system.

### Statistical analysis

Data were checked for normality with the Shapiro–Wilk test. Paired or unpaired t-test was performed for normal continuous variables and the Wilcoxon signed rank test or Mann-Whitney for other variables. Spearman correlation was used for all correlation analysis. Two-way ANOVA with Šidák multiple comparison was used for glucose and insulin tolerance tests. Statistical analyses were performed using Prism, version 8.3.0 (GraphPad Software, US). Data are provided as mean with SEM . P-values <0.05 were considered statistically significant. All authors had access to the study data and reviewed and approved the final manuscript.

### Study approval

The studies were approved by the local Institutional Review Board of the Amsterdam UMC, location AMC in Amsterdam, the Netherlands, and conducted in accordance with the Declaration of Helsinki. Written informed consent was received prior to participation.

## Author contributions

T.P.M.S. performed the experiments and prepared the manuscript. M.W. performed experiments. S.R.H., G.S., M.S. and D.D. assisted with animal experiments. S.M., M.dB., A.vdL. and Ö.A. conducted the BARIA cohort. M.B. and M.D. performed bioinformatic analyses. W.M.dV., C.B., H.Y. and C.M. provided bacterial cultures. M.G.B and O.R.B. provided pancreatic biopsies. G.M.D.T., G.J.B. and W.M.V. aided with the writing. B.J.H.B. conducted the HELIUS cohort. B.A.V., M.N., H.H. and C.B.V. supervised the project. D.H.R. developed the theory and supervised the project.

## Supplementary Material

Supplemental Material

## Data Availability

The data that support the findings of this study are available on request from the corresponding author. The data are not publicly available due to information that could compromise the privacy of research participants and can only be obtained via a data transfer agreement with the HELIUS board. Further information and requests for resources and reagents should be directed to and will be fulfilled by Torsten P.M. Scheithauer. *In vitro* experiments and murine data can be found under the following DOI: 10.4121/20484414.
