# Supplementary material for: Gut-derived bacterial flagellin induces beta-cell inflammation and dysfunction
Source: Gut Microbes. 2022 Aug 19;14(1):2111951. doi: 10.1080/19490976.2022.2111951 (PMC9397137; doi:10.1080/19490976.2022.2111951)
Supplement: Supplemental Material [file KGMI_A_2111951_SM1510.docx]

# Supporting Tables

***Table S1: Characteristics of the Dutch study participant of the HELIUS cohort.***

Supporting information to **Figure 1a**. Data shown are mean ± SD for patient characteristics. Mann Whitney test was used for statistical significance. Abbreviations: ND, no diabetes; T2D, Type 2 diabetes; HbA1c, glycated hemoglobin; n.d., not determined.

|  | **ND** | **T2D** | **p-value** |
| --- | --- | --- | --- |
| n | 712 | 91 | n.d. |
| Sex (%female) | 57 | 34 | <0.0001 |
| Age (years) | 45.8 ± 13.0 | 61.7 ± 5.9 | <0.0001 |
| Body mass index (kg/m^2^) | 23.8 ± 3.5 | 29.2 ± 4.7 | <0.0001 |
| HbA1c (mmol/mol) | 34.3 ± 2.7 | 46.9 ± 8.2 | <0.0001 |

***Table S2: Gut microbiota composition (family level) of the Dutch participants of the HELIUS cohort.***

Supporting information to **Figure 1a**. The 16S rRNA of the fecal microbiota was sequenced via miSeq (family level). More information can be found in Balvers, Deschasaux et al. (2021). Data shown are median (in % of total reads). Abbreviations: ND, no diabetes; T2D, Type 2 diabetes.

| **Bacterial family** | **ND (n = 712)** | **T2D (n = 91)** |
| --- | --- | --- |
| Bacteroidales_Rikenellaceae | 1.62 | 1.22 |
| Bacteroidales_Barnesiellaceae | 0.44 | 0.31 |
| Bacteroidales_Muribaculaceae | 0.54 | 0.67 |
| Bacteroidales_Prevotellaceae | 9.25 | 10.30 |
| Bacteroidales_Bacteroidaceae | 7.90 | 8.20 |
| Bacteroidales_Tannerellaceae | 0.71 | 0.78 |
| Clostridiales_Lachnospiraceae | 30.10 | 31.69 |
| Clostridiales_Peptostreptococcaceae | 1.08 | 0.71 |
| Verrucomicrobiales_Akkermansiaceae | 0.83 | 0.55 |
| Desulfovibrionales_Desulfovibrionaceae | 0.39 | 0.60 |
| Betaproteobacteriales_Burkholderiaceae | 0.68 | 0.74 |
| Enterobacteriales_Enterobacteriaceae | 0.35 | 1.08 |
| Bifidobacteriales_Bifidobacteriaceae | 2.34 | 1.57 |
| Coriobacteriales_Eggerthellaceae | 0.62 | 0.62 |
| Coriobacteriales_Coriobacteriaceae | 1.24 | 1.55 |
| Selenomonadales_Veillonellaceae | 1.74 | 1.92 |
| Selenomonadales_Acidaminococcaceae | 0.88 | 1.16 |
| Erysipelotrichales_Erysipelotrichaceae | 2.06 | 2.66 |
| Lactobacillales_Streptococcaceae | 0.52 | 0.96 |
| Clostridiales_Christensenellaceae | 1.95 | 1.68 |
| Clostridiales_Clostridiaceae_1 | 0.68 | 0.44 |
| Clostridiales_Ruminococcaceae | 30.35 | 27.28 |
| Other | 1.97 | 2.11 |

***Table S3. Characteristics of selected participants from the HELIUS cohort.***

Supporting information for **Figure 1b-e**. Participants were randomly selected from the HELIUS cohort. Participants with Type 2 diabetes (T2D) were age-sex-BMI matched to normoglycemic (ND) controls. Data shown are mean ± SD. Unpaired t-test was used for age and BMI. Mann Whitney test for fasting glucose and HbA1c. Abbreviations: BMI, body mass index; HbA1c, Glycated hemoglobin.

|  | **ND (n = 50)** | **T2D (n = 100)** | **p-value** |
| --- | --- | --- | --- |
| Age (years) | 57.2 ± 6.8 | 56.8 ± 6.7 | 0.7440 |
| Female (%) | 62 | 62 | 1.0000 |
| BMI (kg/m^2^) | 29.7 ± 4.4 | 29.8 ± 5.0 | 0.9673 |
| Fasting glucose (mmol/L) | 5.35 ± 0.56 | 7.13 ± 1.85 | <0.0001 |
| HbA1c (mmols/mol) | 40.0 ± 4.2 | 54.5 ± 15.2 | <0.0001 |

***Table S4. Characteristics of selected participants from BARIA cohort.***

Participants were randomly selected from BARIA cohort. People with Type 2 diabetes (T2D) were matched to controls without T2D (ND) according to age, sex and body mass index (BMI). Baseline samples were used before bariatric surgery. Data shown are mean ± SD. Unpaired t-test was used. Abbreviations: n.d., not determined; BMI, body mass index; HbA1c, Glycated hemoglobin.

|  | **ND (n = 40)** | **T2D (n = 40)** | **p-value** |
| --- | --- | --- | --- |
| Age (years) | 49.2 ± 9.9 | 49.4 ± 10.2 | 0.9468 |
| Female (%) | 65 | 65 | 1.000 |
| BMI (kg/m^2^) | 39.4 ± 3.4 | 39.2 ± 4.7 | 0.8666 |
| Fasting glucose (mmol/L) | 5.7 ± 0.8 | 7.3 ± 1.6 | <0.0001 |
| HbA1c (%) | 5.7 ± 0.4 | 7.3 ± 1.1 | <0.0001 |
| C-peptide (nmol/L) | 0.92 ± 0.3 | 0.94 ± 0.4 | 0.7463 |
| Insulin (pmol/L) | 91.3 ± 46.0 | 189.1 ± 239.5 | 0.0144 |

## Table S5. Characteristics of individuals that underwent pancreatic surgery.

Individuals who are scheduled for pancreatic surgery (e.g., pylorus-preserving pancreatoduodenectomy or Whipple’s procedure), because of pancreatic carcinoma, were asked to donate healthy tissue surrounding the tumor. Antibodies against flagellin were measured in homogenized samples. Abbreviations: T2D, Type 2 diabetes; M, male: F, female: BMI, body mass index: HbA1c, glycated hemoglobin.

|  | **T2D (n = 5)** |
| --- | --- |
| Age (years) | 49.4 ± 10.2 |
| Sex (M/F) | 3/2 |
| BMI (kg/m^2^) | 27.1 ± 3.0 |
| HbA1c (%) | 7.8 ± 1.4 |
| Diabetes duration (years) | 9 ± 5 |

***Table S6. Primer sequences used in this manuscript (both in 5´3´direction).***

| **Name** | **Species** | **Forward** | **Reverse** | **Ref.** |
| --- | --- | --- | --- | --- |
| Eclo_V3V4 | *Enterobacter cloacae* | CAGCAATTGACGTTACCCGC | CAGCCTGCCAGTTTCGAATG | This study |
| En-lsu3 | Enterobacteriaceae | TGCCGTAACTTCGGGAGAAGG | TCAAGGCTCAATGTTCAGTGTC | PMID 17071791 |
| EUBAC | Bacteria | TCCTACGGGAGGCAGCAGT | GGACTACCAGGGTATCTAATCCTGTT | PMID 11782518 |
| RPLP0 | Human | ACGGGTACAAACGAGTCCTG | GCCTTGACCTTTTCAGCAAG | This study |
| RPLP0 | Rat | GAACATCTCCCCCTTCTCCTTC | ATTGCGGACACCCTCTAGGAA | This study |
| Rps18 | Mouse | CAC TTT TGG GGC CTT CGT G | GCA AAG GCC CAG AGA CTC ATT | This study |
| NLRP3 | Mouse | AGA GCC TAC AGT TGG GTG AA | CTT CCA ACG CCT ACC AGG AAA T | This study |
| NLRP3 | Human | CAGAACCTGGGGTTGTCTGAA | GAAGGCTCAAAGACGACGGT | This study |
| MafA | Human | GAGAGCGAGAAGTGCCAACT | CTTGTACAGGTCCCGCTCTTT | This study |
| MafA | Mouse | CAA GGA GGA GGT CAT CCG AC | TCT CCA GAA TGT GCC GCT G | This study |
| MafA | Rat | GCACCCGACTTCTTTCTGTGA | GCCTCAGAGTCCGAACCGA | This study |
| PDX1 | Human | AAA GCT CAC GCG TGG AAA G | GGC CGT GAG ATG TAC TTG TTG | This study |
| PDX1 | Mouse | CAG TGG GCA GGA GGT GCT TA | GGG CCG GGA GAT GTA TTT GTT | This study |
| PDX1 | Rat | TTCATCTCCCTTTCCCGTGG | GTGTAGGCTGTACGGGTCCT | This study |
| INS1 | Mouse | GAC CAT CAG CAA GCA GGT CAT T | GAC AAA AGC CTG GGT GGG TT | This study |
| INS1 | Rat | CACACCCAAGTCCCGTCGT | AACCTCCAGTGCCAAGGTCTG | This study |
| INS | Human | TCT ACC TAG TGT GCG GGG AA | TCC ACC TGC CCC ACC TG | This study |
| INS2 | Mouse | AGG CTC TCT ACC TGG TGT GT | TCT GAA GGT CAC CTG CTC CC | This study |
| INS2 | Rat | AACCATCAGCAAGCAGGTCA | TCCACCAAGTGAGAACCACA | This study |
| Glut2 | Mouse | AATGGTCGCCTCATTCTTTG | AGCCAACATTGCTTTGATCC | This study |
| Glut2 | Rat | TTGCTCCAACCACACTCAGG | CTGAGGCCAGCAATCTGACT | This study |
| Glut2 | Human | TGCCACACTCACACAAGACC | AACTGGAAGGAACCCAGCAC | This study |
| F4/80 | Mouse | TGACAACCAGACGGCTTGTG | GCAGGCGAGGAAAAGATAGTGT | This study |
| CD68 | Human | CCC CAA CAA AAC CAA GGT CC | GGA GGT CCT GCA TGA ATC CAA A | This study |
| TNF-α | Mouse | CTGTAGCCCACGTCGTAGC | TTGAGATCCATGCCGTTG | This study |
| TNF-α | Human | CAGCCTCTTCTCCTTCCTGAT | GCCAGAGGGCTGATTAGAGA | This study |
| IL-1β | Mouse | GCAACTGTTCCTGAACTCAACT | ATCTTTTGGGGTCCGTCAACT | This study |
| IL-1β | Rat | TTTCGACAGTGAGGAGAATGACC | CTGGACAGCCCAAGTCAAGG | This study |
| IL-1β | Human | GCT GAG GAA GAT GCT GGT TC | GTG ATC GTA CAG GTG CAT CG | This study |
| IL-6 | Mouse | TCGTGGAAATGAGAAAAGAGTTGTG | TCCAGTTTGGTAGCATCCATCAT | This study |
| TLR2 | Mouse | TAGGGGCTTCACTTCTCTGC | CCAAAGAGCTCGTAGCATCC | This study |
| TLR5 | Mouse | CTGGAGCCGAGTGAGGTC | CGGCAAGCATTGTTCTCC | This study |
| TLR5 | Human | GAC ACA ATC TCG GCT GAC TG | TCA GGA ACA TGA ACA TCA ATC TG | This study |
| TLR5 | Rat | GACCCAGTATGCTCGCTTGA | GATGGGGCAGTCCCTGAAAA | This study |

|  | **1** | **2** | **3** |
| --- | --- | --- | --- |
| **Supplier** | Prodo Labs | Prodo Labs | Prodo Labs |
| **Sex** | Male | Male | Female |
| **Age** | 38 | 38 | 61 |
| **Ethnicity** | Caucasian | Hispanic | Caucasian |
| **BMI** | 24.1 | 28 | 31.9 |
| **HbA1c (%)** | 5.8 | 5.9 | 5.8 |
| **Cause of death** | Anoxic event | Head trauma | Stroke |
| **Purity (%)** | 90 | 90 | 90 |
| **Viability (%)** | 95 | 95 | 95 |

***Table S7. Characteristics of donors of human islets used in this manuscript.***


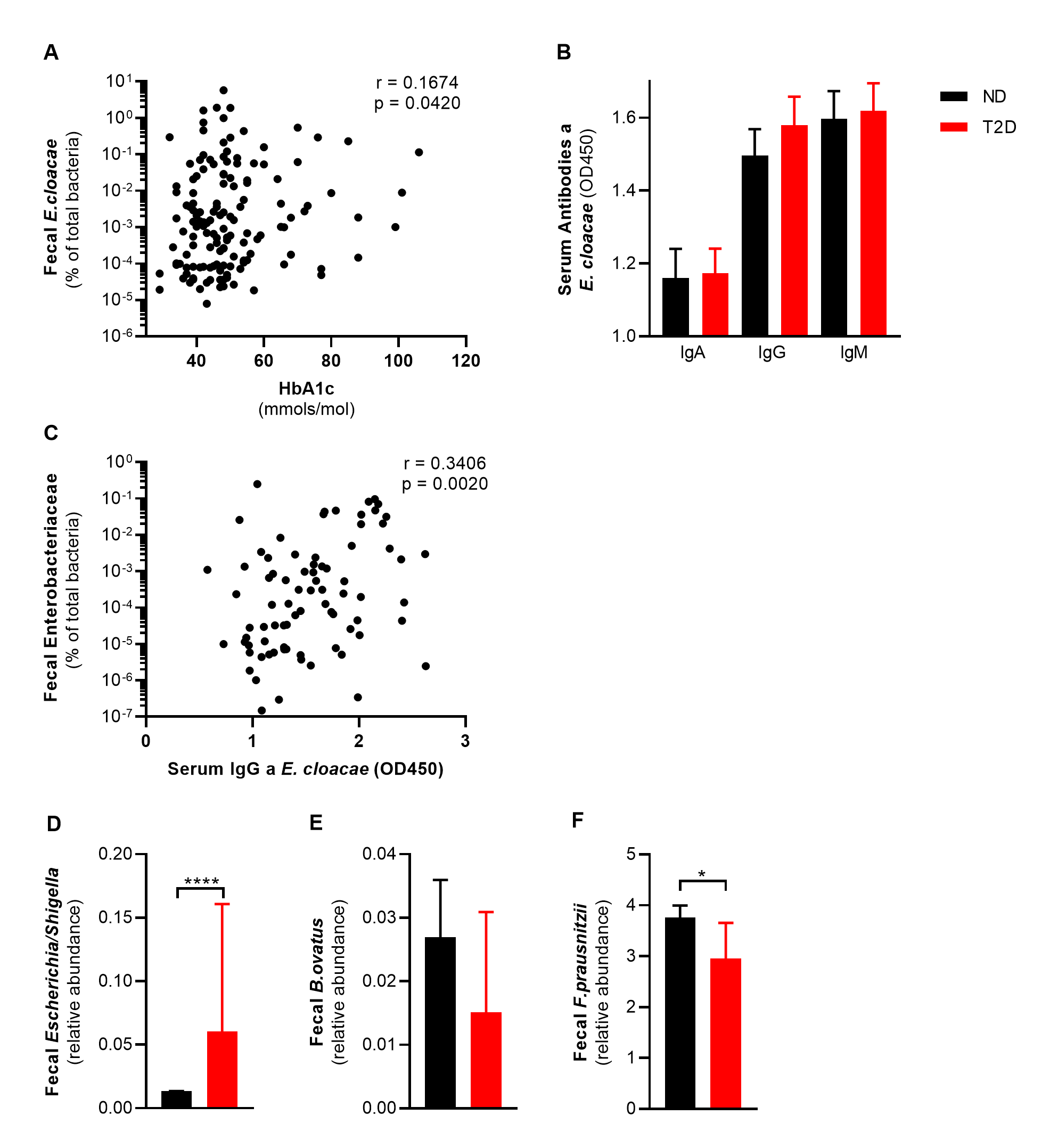


***Figure S1. Fecal pathogens are associated with glucose intolerance.***

(A) Fecal *Enterobacter cloacae* positively correlates with glucose marker HbA1c in a subset of Dutch origin participants of the HELIUS cohort (N = 150).

(B) Serum antibodies against *E. cloacae* are not different between ND and T2D in subset of Dutch origin participants of the HELIUS cohort (N = 80).

(C) Serum IgG anti *E.cloacae* positively correlates with fecal Enterobacteriaceae (N = 80, Spearman correlation).

(D) Fecal *Escherichia* is increased in T2D (HELIUS cohort, N = 803)

(E) Fecal *Bacteroides ovatus* is non-significantly decreased in T2D (HELIUS cohort, N = 803, mean with 95% confidence interval)

(F) Fecal *Fecalibacterium prausnitzii* is decreased in T2D (HELIUS cohort, N = 803)

Data shown are mean ± SEM, except for gut microbiota (median with 95% confidence interval). Spearman correlation (A, C) and Mann Whitney test (D, F) was used: *p<0.05, ****p<0.0001. Abbreviations: ND, no diabetes; T2D, Type 2 diabetes.


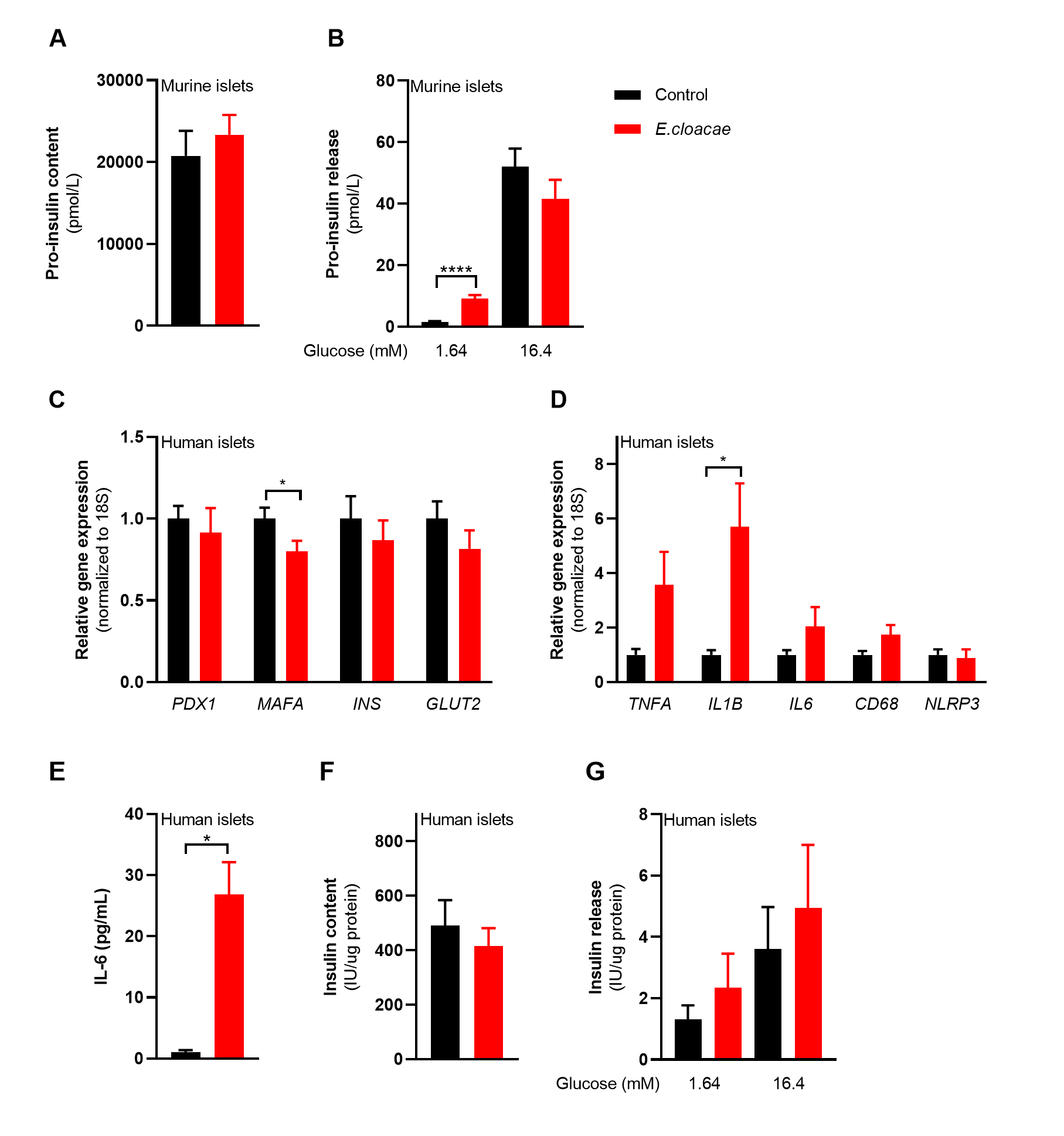


***Figure S2. E.cloacae induces beta-cell dysfunction in pancreatic islets.***

Murine pancreatic islets were treated with heat-inactivated *E. cloacae* for 72h. Pro-insulin as well as insulin was measured during a glucose stimulated insulin secretion assay (A, B). Human islets were ordered from ProdoLabs and treated with *E.cloacae* (1E6 CFUs/mL) for 72h (C-G).

(A) *E.cloacae* non-significantly increase pro-insulin content in murine pancreatic islets.

(B) *E.cloacae* increased pro-insulin release from murine pancreatic islets during basal glucose conditions.

(C) *E.cloacae* reduces beta-cell marker expression in human islets.

(D) *E.cloacae* induces beta-cell inflammation in human islets.

(E) *E.cloacae* induces IL-6 release from human islets.

(F) *E.cloacae* slightly reduces insulin content in human islets.

(G) *E.cloacae* slightly induces insulin hypersecretion in human islets.

Data shown are mean ± SEM (3 representative experiments A-B; 2 experiments C-G). Unpaired t-test was used for statistical analysis: *p<0.05, **p<0.01, ***p<0.001, ****p<0.0001.

Abbreviations: INS1 and INS2, insulin 1 and 2; NLRP3, NACHT, LRR and PYD domains-containing protein 3; IL-1β, Interleukin 1 beta; IL-6, Interleukin 6; TLR, toll like receptor.


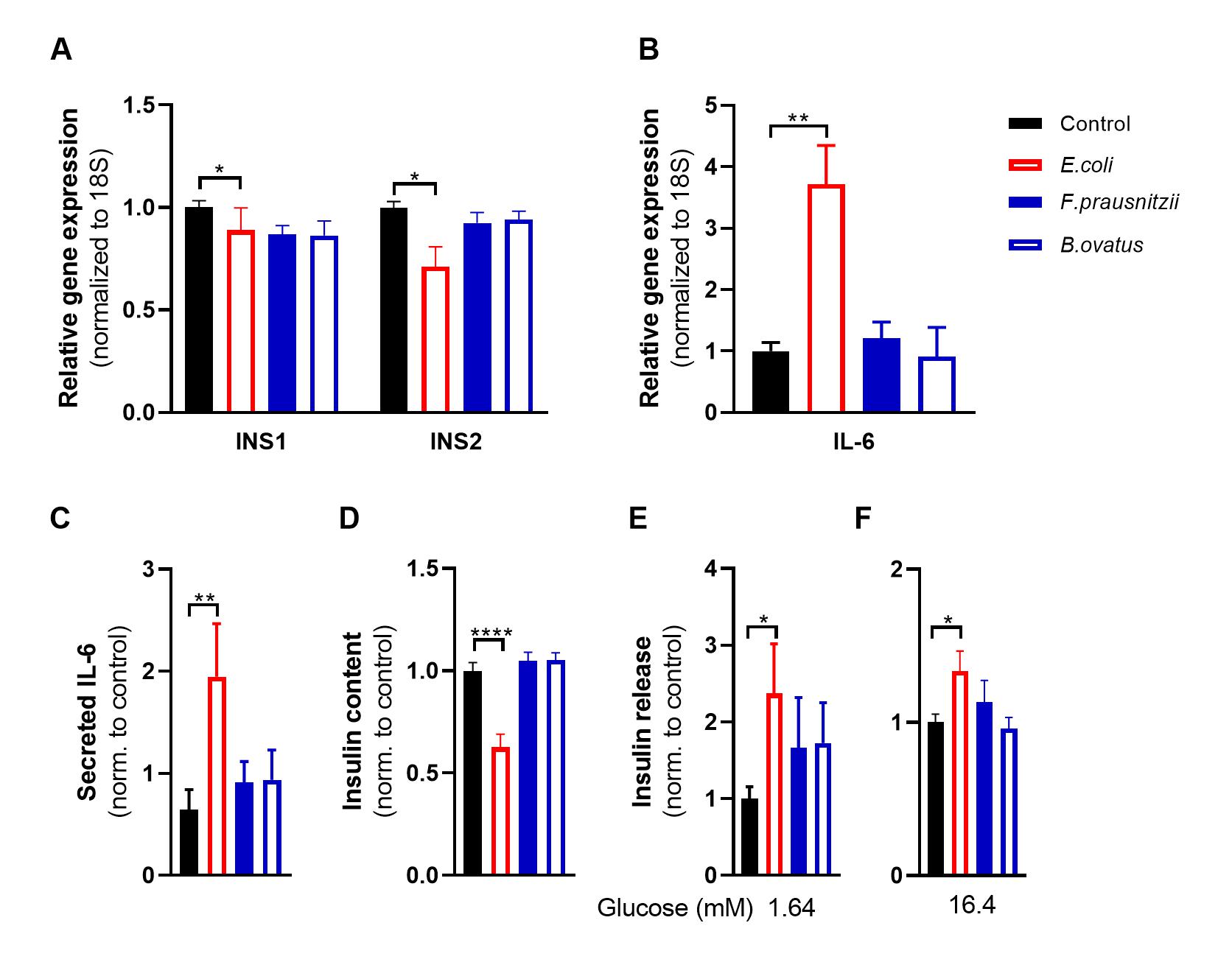


### **Figure S3. Opportunistic pathogens, but not beneficial bacteria induce beta-cell inflammation and dysfunction**

Freshly isolated pancreatic islets from healthy C57BL6J mice were treated with heat-inactivated bacteria for 72h (10^6^ colony forming units/mL).

(A) *Escherichia coli*, but not *Faecalibacterium prausnitzii and Bacteroides ovatus*, reduces expression of insulin genes.

(B) *E. coli*, but not *F. prausnitzii and B. ovatus* increases expression of IL-6 in islets.

(C) *E. coli*, but not *F. prausnitzii and B. ovatus*, induces the release of IL-6 from islets into the media.

(D) *E. coli*, but not *F. prausnitzii and B. ovatus* reduces insulin content in islets.

(E) *E. coli*, but not *F. prausnitzii and B. ovatus* induces insulin hypersecretion versus controls at low-glucose conditions.

(F) *E. coli*, but not *F. prausnitzii and B. ovatus* induces insulin hypersecretion versus controls at high-glucose conditions.

Data shown are mean ± SEM (3 representative experiments per panel). Unpaired t-test (A, B, C, D) and Mann Whitney test (E, F) was used. Significance level: *p<0.05, **p<0.01, ****p<0.0001. Gene expression was normalized using *18s* as a reference gene. Panels C-F were normalized to the control samples since the experiments were performed per bacteria. Abbreviations: INS1 and INS2, insulin 1 and 2; IL-6, Interleukin 6.

**Figure S4. TLR2 and TLR4 knock out do not protect from beta-cell dysfunction**

Freshly isolated pancreatic islets from C57BL6J TLR2^-/-^ (A-E) and TLR4^-/-^ (F-J) mice were treated with heat-inactivated *Enterobacter cloacae* (10^6^ CFUs/mL) for 72h.

(A) *E. cloacae* reduces expression of insulin genes both in wild-type and TLR2 knock out pancreatic islets of C57BL6J mice.

(B) *E. cloacae* increases expression of IL-6 in wild-type pancreatic islets of C57BL6J mice.

(C) *E. cloacae* increases secreted IL-6 from by wild-type and TLR2 knock out pancreatic islets of C57BL6J mice.

(D) *E. cloacae* reduces insulin content in wild-type and TLR2 knock out pancreatic islets of C57BL6J mice.

(E) *E. cloacae* induces insulin hypersecretion in wild-type and TLR2 knock out pancreatic islets during low-glucose concentrations of C57BL6J mice.

(F) *E. cloacae* reduces expression of insulin genes both in wild-type and TLR4 knock out pancreatic islets of C57BL6J mice.

(G) *E. cloacae* increase expression of IL-6 in islets in wild-type pancreatic islets of C57BL6J mice.

(H) *E. cloacae* increases secreted IL-6 from by wild-type pancreatic islets of C57BL6J mice.

(I) *E. cloacae* reduces insulin content in wild-type and TLR4 knock out pancreatic islets of C57BL6J mice.

(J) *E. cloacae* induces insulin hypersecretion at low glucose concentrations in wild-type and TLR4 knock out pancreatic islets of C57BL6J mice.

Data shown are mean ± SEM (3 representative experiments per panel). Unpaired t-test (A-J) was used for statistical analysis. Abbreviations: INS1 and INS2, insulin 1 and 2; IL-6, Interleukin 6; TLR, Toll-like receptor.


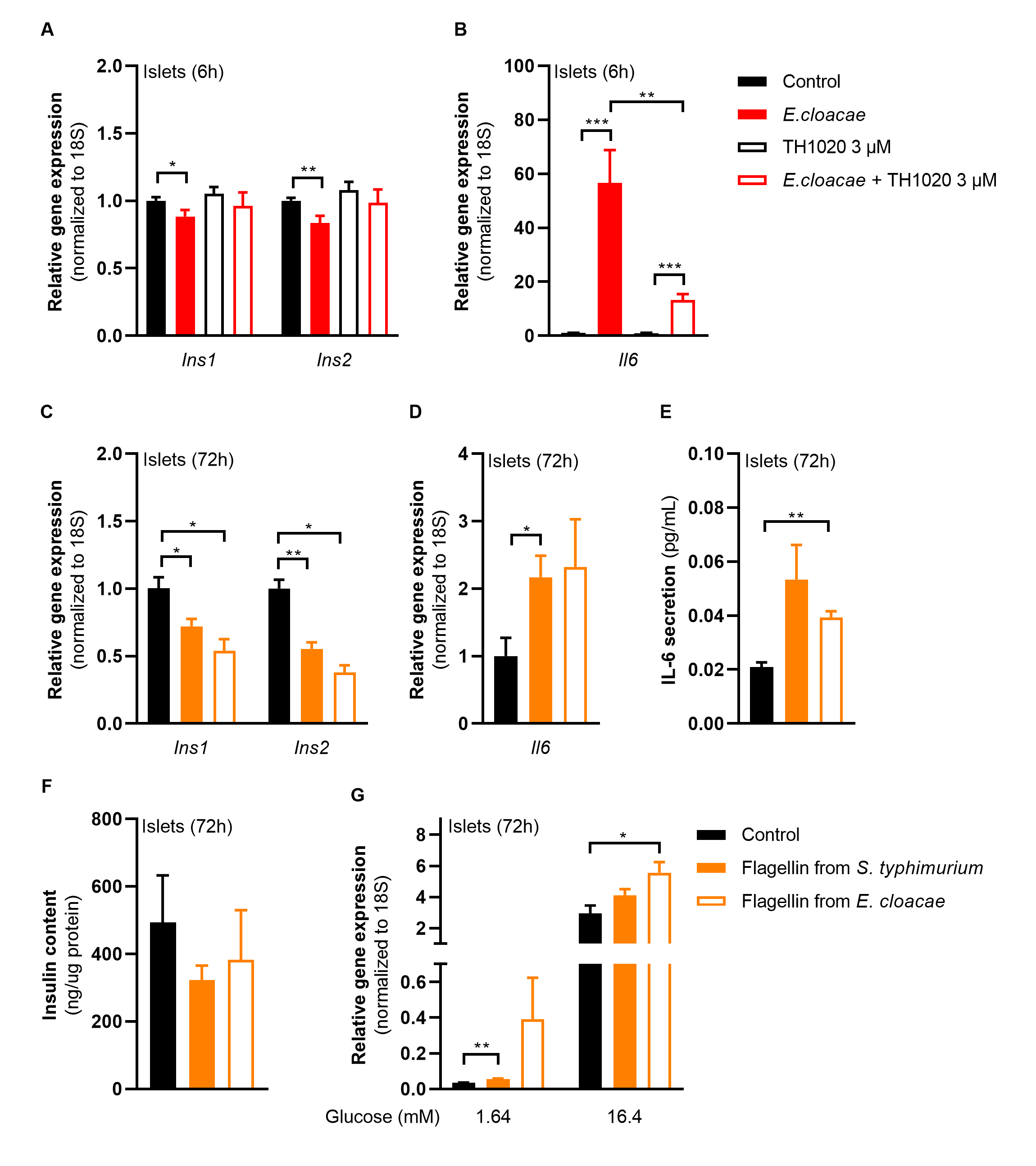


***Figure S5. TLR5 inhibitor reduces bacteria induced beta-cell dysfunction.***

Pancreatic islets were isolated from C57BL6J mice and incubated with TLR5 inhibitor TH1020 (3 uM) and *E.cloacae* (10^6^ CFUs/mL) for 6h (A-B). Freshly isolated pancreatic isolates were either incubated with flagellin from *Salmonella typhimurium* or from *E.cloacae* for 72h (C-G).

(A) TLR5 inhibitor TH1020 reverses bacteria induced pancreatic islet dysfunction (6h incubation).

(B) TLR5 inhibitor TH1020 reduces bacteria induced pancreatic islet inflammation (6h incubation).

(C) Flagellin reduces insulin expression in pancreatic islets.

(D) Flagellin induces IL6 expression in pancreatic islets.

(E) Flagellin induces IL6 secretion from pancreatic islets.

(F) Flagellin non-significantly reduces insulin content in pancreatic islets.

(G) Flagellin induces insulin hypersecretion in pancreatic islets.

Data shown are mean ± SEM (3 representative experiments for A-B; 1 representative experiment for C-G). Unpaired t-test was used for statistical analysis: *p<0.05, **p<0.01, ***p<0.00.

Abbreviations: INS1 and INS2, insulin 1 and 2; IL-6, Interleukin 6.


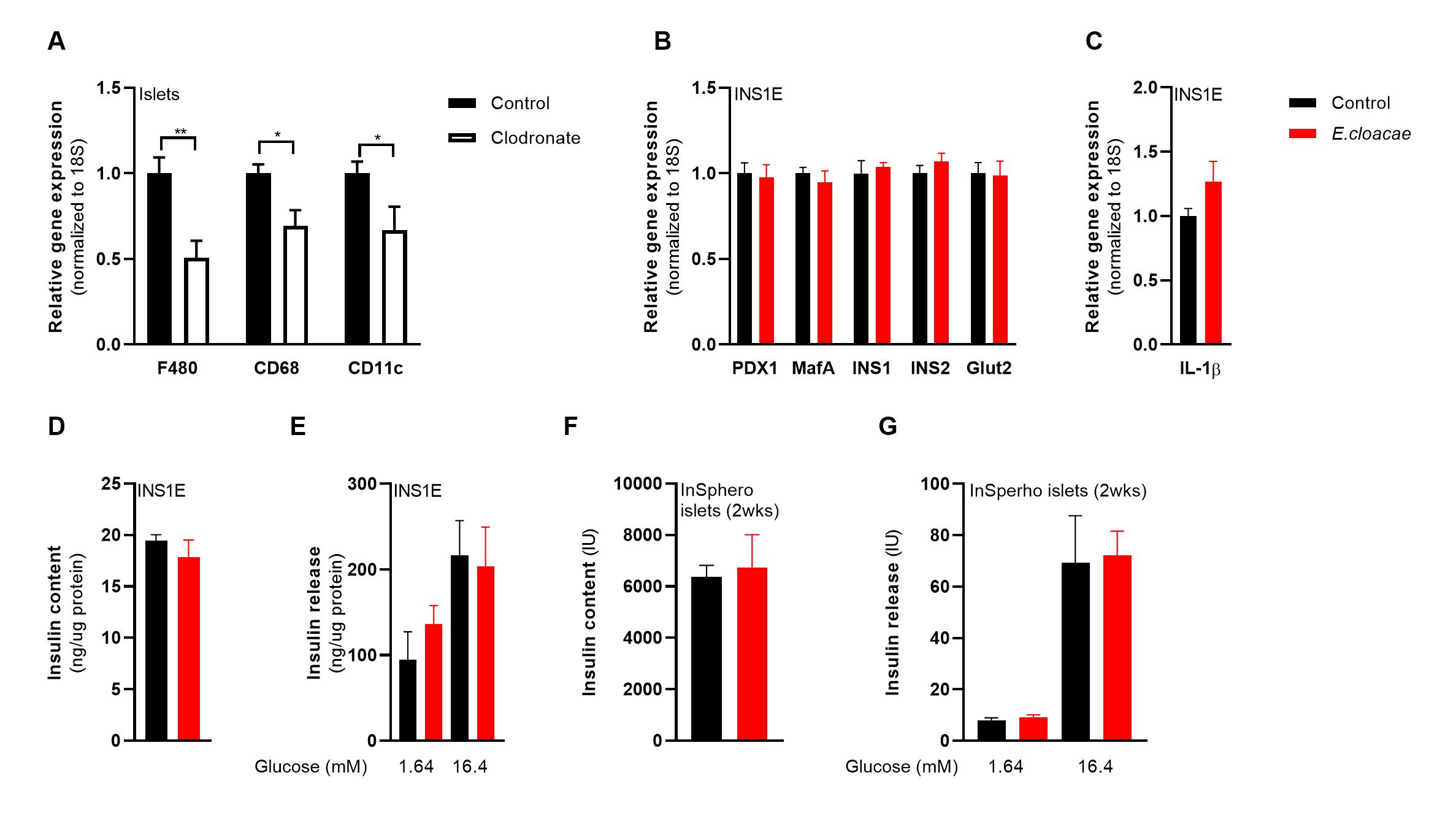


***Figure S6. Clonal beta cells are not affected by*** *E.cloacae* treatment***.***

Murine pancreatic islets were treated with Clodronate-liposome for 48h (A). with Pure beta cells (INS1E, B-E) and modified human islets without immune cells (InSphero islets, F-G) were treated with *E.cloacae* for 72h.

(A) Clodronate-liposome reduces macrophage expression in murine pancreatic islets.

*(B) E.cloacae* does not reduce beta-cell marker expression.

(C) *E.cloacae* does not induce beta-cell inflammation.

(D) *E.cloacae* does not reduce insulin content in beta cells.

(E) *E.cloacae* does not induce insulin hypersecretion in beta cells.

(F) *E.cloacae* does not reduce insulin content in InSphero islets.

(G) *E.cloacae* does not induce insulin hypersecretion in InSphero islets.

Data shown are mean ± SEM (3 representative experiments). Unpaired t-test was used for statistical analysis: *p<0.05, **p<0.01, ***p<0.001, ****p<0.0001.

Abbreviations: INS1 and INS2, insulin 1 and 2; IL-1β, Interleukin 1 beta.


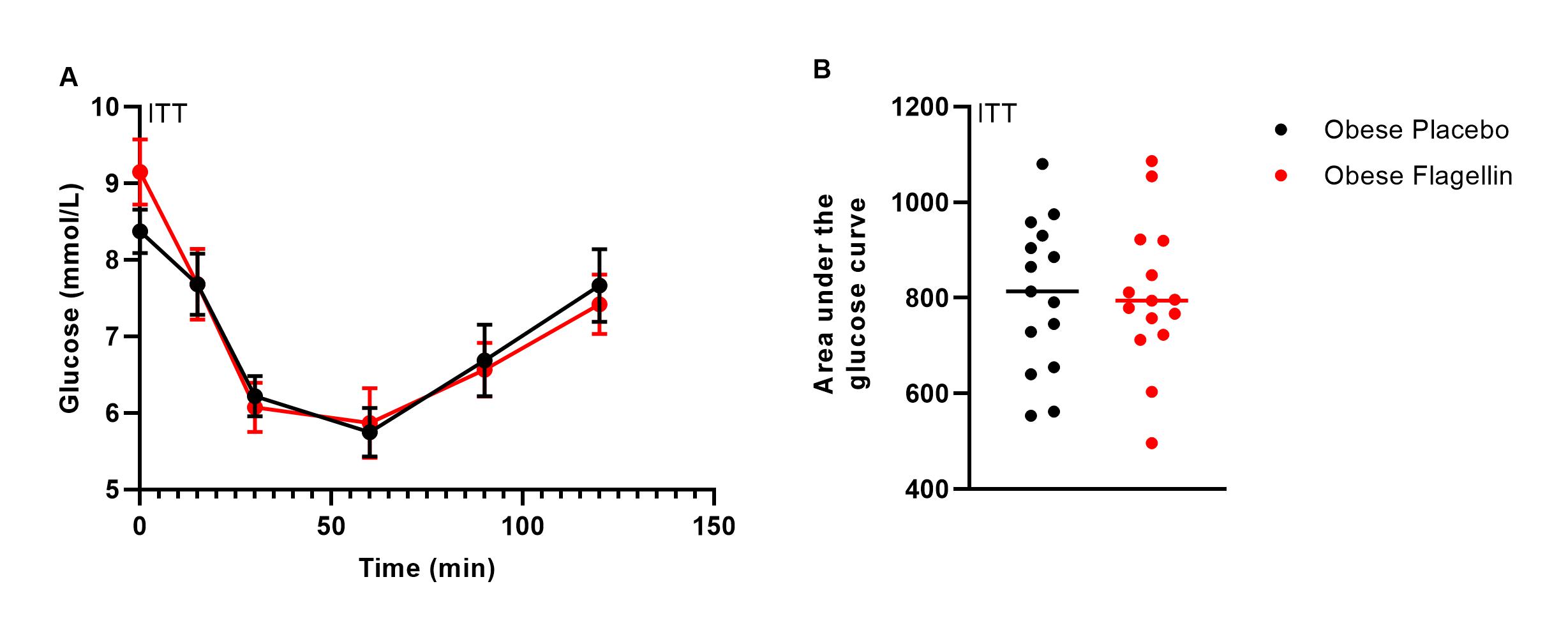


***Figure S7. Flagellin injection does not affect insulin tolerance in mice***

Six-week old mice were fed a high fat diet (60%kcal fat) for 12 weeks. In the last 4 weeks of the diet, the mice were injected with either 1 µg flagellin in 100 µL saline or saline alone twice weekly.


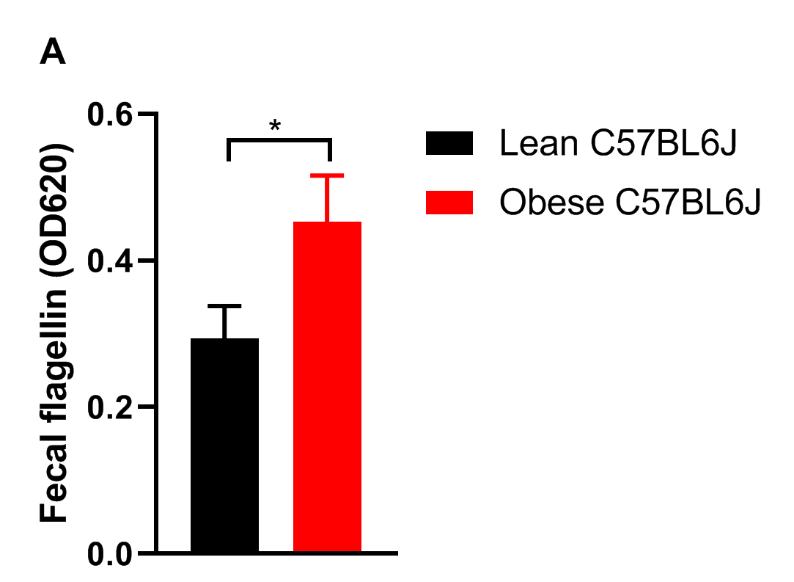


***Figure S8. High fat diet feeding increases fecal flagellin content in mice.***

C57BL6J mice were on a high fat diet (60% kcal fat) for 12 weeks. Fecal flagellin was measured in homogenized samples with HEK TLR5 reporter cells.


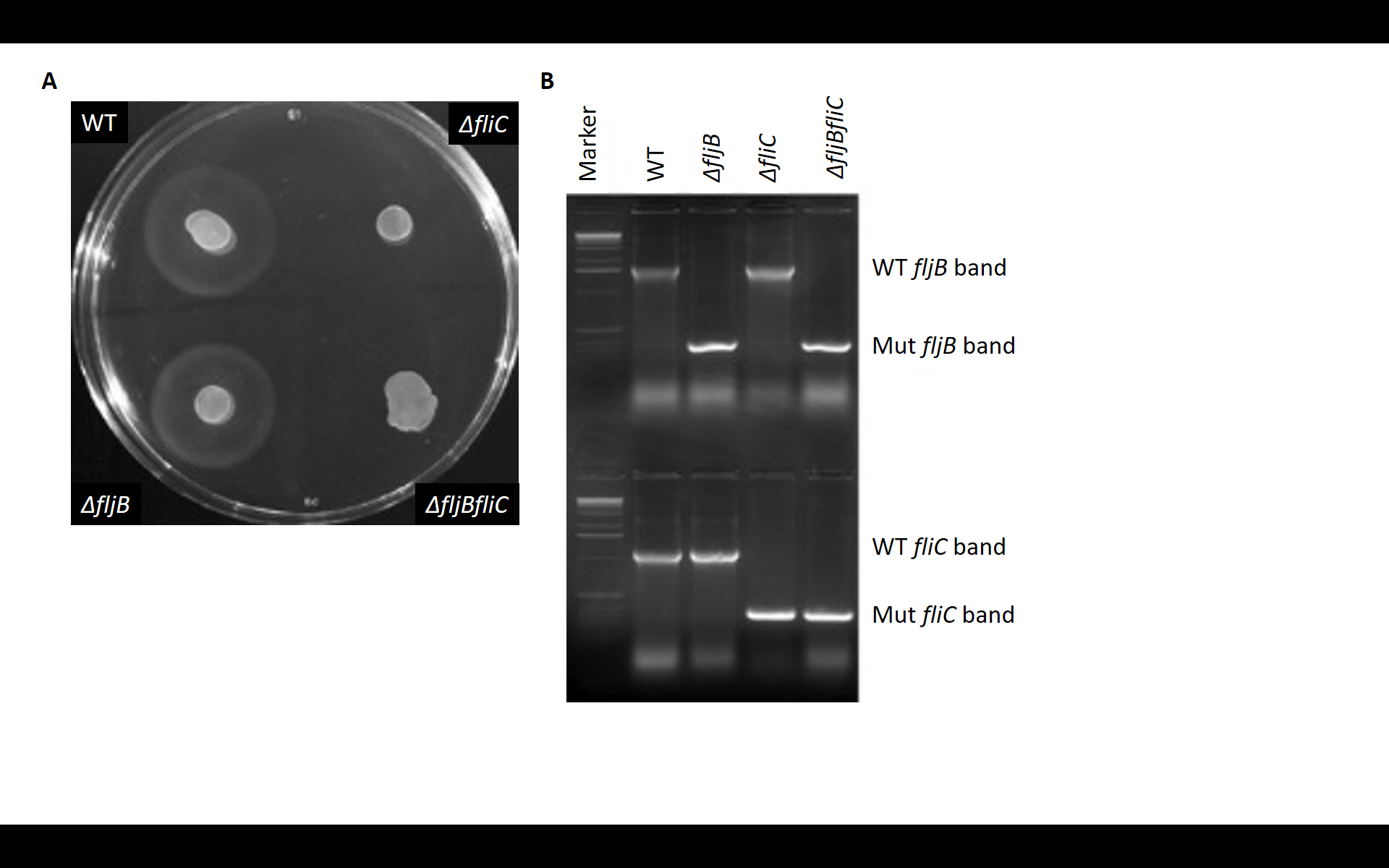


**Figure S9: Generation of flagellin depleted *E.cloacae*.**

Supporting data for methods. The flagellin genes fliB and fliC were knocked out in *E.cloacae*. Abbreviations: WT, wild-type; Mut, mutant; Fli, flagellin gene B or C.
